# A Paired Ileum–Caecum Study of the Goose Gut Microbiome in Response to Fattening Protocol and Postbiotic Supplementation

**DOI:** 10.3390/ani16193153

**Published:** 2026-10-08

**Authors:** Erzsébet Baka, Mária Kovács-Weber, Renáta Ábrahám, Tibor István Pap, András Táncsics, Gyula Szabó, Mónika Heincinger, Stella Szabó, Ágnes Dolányi, Andrea Ilona Berta, Balázs Kovács, Rubina Tünde Szabó

**Affiliations:** 1 Institute of Aquaculture and Environmental Safety, Hungarian University of Agriculture and Life Sciences, H-2100 Gödöllő, Hungary; baka.erzsebet@uni-mate.hu (E.B.); abraham.renata@uni-mate.hu (R.Á.); tancsics.andras@uni-mate.hu (A.T.); gyula.szabo.mate@gmail.com (G.S.); 2 Institute of Animal Husbandry, Gödöllő Campus, Hungarian University of Agriculture and Life Sciences, H-2100 Gödöllő, Hungary; kovacs-weber.maria@uni-mate.hu (M.K.-W.); pap.tibor.istvan@uni-mate.hu (T.I.P.); szabostellahun@gmail.com (S.S.); berta.andrea.ilona@phd.uni-mate.hu (A.I.B.); szabo.rubina.tunde@uni-mate.hu (R.T.S.); 3 Lab-Nyúl Ltd., Malomtó u. 8., H-2100 Gödöllő, Hungary; heincinger.monika@gmail.com (M.H.); agi.dolanyi@gmail.com (Á.D.)

**Keywords:** goose microbiota, 16S rRNA gene sequencing, postbiotic, fattening protocol, ileum, caecum, microbial diversity

## Abstract

The gut microbiota plays a central role in poultry health and production performance, yet its response to intensive fattening protocols and postbiotic supplementation in geese remains poorly characterized. This study used 16S rRNA gene amplicon sequencing to profile ileal and caecal microbial communities from geese raised under two fattening protocols, with or without postbiotic supplementation, and assessed the effect of 24 h post-mortem chilling on apparent community composition. Fattening protocol was associated with the largest differences in gut microbial structure, producing significant shifts in beta diversity in both the ileum and the caecum (although protocol, age at slaughter, and duration of postbiotic exposure co-varied between cohorts and cannot be fully disentangled), accompanied by extensive taxon-level differences identified by LEfSe. Postbiotic supplementation did not significantly alter overall community structure under force-feeding conditions but produced a taxon-specific, trend-level shift in the caecum under restricted feeding, suggesting that its effect depends on the continuity of supplementation relative to slaughter. Alpha diversity did not differ significantly in the ileum, but caecal richness differed significantly between the G2 and G5 postbiotic-supplemented groups (Conover–Iman test, adjusted *p* < 0.05). The warm- and chilled-dissection groups differed in ileal but not caecal community composition (individual-animal variation cannot be separated from the effect of chilling in this comparison, as the two groups consisted of different birds), indicating that dissection timing is an important methodological variable in goose microbiome research. These findings highlight cohort- and protocol-related differences as key correlates of gut microbial composition in geese and provide a framework for optimizing postbiotic supplementation strategies and sampling standardization in waterfowl production.

## Simple Summary

Geese raised for fattened liver production undergo intensive feeding regimes that can disrupt gut health, prompting interest in dietary postbiotics as a supportive intervention. This study examined the small and large intestinal bacterial communities of geese raised under two different fattening protocols, with and without a commercial postbiotic supplement (Burain^®^), and additionally tested whether delaying sample collection after slaughter (24 h chilling) affects the observed gut microbiota. Using 16S rRNA gene sequencing of 38 intestinal samples, we found that the fattening protocol itself had a far greater influence on gut bacterial composition than the postbiotic supplement, particularly in the caecum. Group sizes were small (n = 3–4 per group and intestinal segment), and protocol-related differences could not be fully separated from cohort effects. The postbiotic produced detectable but more subtle shifts in specific bacterial groups, which were more apparent when supplementation continued up to slaughter than when it had been discontinued earlier. We also found that the warm- and chilled-dissection groups differed in their bacterial community of the small intestine, but not the caecum—an exploratory comparison, since the two groups consisted of different individual birds of the same age, highlighting an important methodological consideration for future goose microbiome studies. These findings help clarify how production practices shape the gut microbiome in geese and support the refinement of postbiotic strategies and sampling protocols in waterfowl fattening systems.

## 1. Introduction

Geese (*Anser anser domesticus*) are economically important herbivorous waterfowl valued for meat, feathers, and high-value fattened liver (foie gras). Their unique digestive physiology supports efficient fiber utilization and marked hepatic fat deposition, making them an attractive model for studying interactions between nutrition, gut microbiota, and liver metabolism. The gastrointestinal microbiome is increasingly recognized as a key determinant of nutrient utilization, immune homeostasis, and metabolic health in geese, yet detailed information on segment-specific communities, particularly in the ileum and caecum, remains scarce [1,2].

The goose gut microbiota is strongly compartmentalized along the gastrointestinal tract. A survey across multiple intestinal segments identified 29 phyla and 359 genera, with the caecum harbouring markedly higher Bacteroidetes and Fusobacteria than other sections, dominated by *Bacteroides*, *Faecalibacterium*, and *Butyricicoccus* in *Anser cygnoides* [1]. Consistent with this composition, acetic and butyric acid concentrations are highest in the caecum, and branched-chain short-chain fatty acids (SCFAs) are detectable only in this segment, confirming its central role in fiber fermentation and energy harvest [1]. In Changle geese (*Anser cygnoides*), integrated histomorphology and 16S rRNA gene profiling confirmed well-developed caecal compartments with distinct regional microbiota, and suggested underappreciated contributions of non-caecal communities to digestion and immune regulation [2]. In contrast, the ileum, as the terminal small intestinal segment, is a key site for nutrient absorption, bile acid reuptake, and interaction with dietary and postbiotic interventions, yet its microbiota remains far less characterized than that of the caecum, and explicit ileum–caecum comparisons in production settings remain limited [1,3].

The goose gut microbiome of *Anser anser* is highly responsive to dietary and management factors. Fasting reduces alpha and beta diversity and shifts phylum-level composition toward metabolic profiles consistent with increased fatty acid oxidation [4]. Overfeeding induces dysbiosis characterized by enrichment of *Escherichia–Shigella* and depletion of specific *Clostridia*, coupled to disrupted bile acid profiles and hepatic lipid accumulation along a gut microbiota–bile acid–liver axis [5,6]. Force-feeding also alters intestinal morphology in *Anser cygnoides*, increasing villus height and small intestinal length while shifting Enterobacteriaceae-related signals, with breed-specific differences in gut stability and liver fat deposition [7]. Housing systems further modulate caecal communities: cage rearing enriches inflammation-related genera while floor rearing favours beneficial lactobacilli in the ileum, with corresponding differences in foie gras capacity [3,8]. Body weight performance in geese (*Anser cygnoides)* has also been correlated with caecal microbiota composition, with *Prevotellaceae_Ga6A1_group* positively associated with leg and breast muscle development [9]. Collectively, these findings position the goose gut microbiome as a central mediator of nutritional strategies and production outcomes, yet most existing work focuses on caecal or fecal samples [1].

Postbiotics, defined as preparations of inactivated microorganisms or their components that confer a health benefit on the host [10], have emerged as promising alternatives to antibiotic growth promoters in poultry production. Postbiotic compounds can inhibit pathogens including *Escherichia coli*, *Salmonella typhimurium*, *Clostridium perfringens*, and *Listeria monocytogenes*, and have been shown to improve growth performance, gut morphology, and immune function in broiler chickens [11,12,13]. In geese, postbiotic research is still in its early stages. Our previous study was the first to evaluate the effect of Burain^®^ postbiotic lysates, derived from *Lactobacillus reuteri*, *L. acidophilus*, and *Bifidobacterium longum*, on production parameters in rearing geese, *A. anser* [14]. Supplementation at 2 g/kg significantly increased body weight at week 8 and reduced the feed conversion ratio, without negative effects on foie gras weight or breast and thigh weights, while liver colour parameters were modified, suggesting that rearing-phase postbiotic effects can persist into the overfeeding period [14]. That study explicitly highlighted the need to evaluate postbiotic effects on gut microbiome structure and to connect rearing-phase interventions to subsequent fattening performance [14].

Beyond nutritional interventions, microbiome-based studies in poultry are also sensitive to methodological factors at the point of sample collection. Post-mortem changes in intestinal microbial communities are well documented: in caecal samples from human autopsies, *Bacteroides* and *Lactobacillus* abundances changed significantly within the first days post-mortem even under refrigeration conditions [15]. In the context of commercial poultry processing, immersion chilling has been shown to dramatically reduce microbiome diversity, with only approximately 10% of OTUs detectable post-chill [16]. More broadly, storage temperature and the time elapsed between collection and freezing can alter apparent microbial community composition as detected by 16S rRNA amplicon sequencing [17,18]. In commercial goose production, warm carcass sampling immediately after slaughter and chilled carcass sampling following 24 h refrigeration represent two practically relevant but microbiologically distinct scenarios, yet their equivalence for intestinal microbiome profiling has not been formally evaluated in geese. Addressing this question has direct practical relevance for the standardization and cross-study comparability of goose microbiome data.

The present study was conducted partially on the same animals as Szabó et al. [14], providing an integrated dataset linking gut microbiome structure to production performance. Two slaughter groups were established: a December group subjected to 14 days of force-feeding without postbiotic supplementation following an 8-week rearing period, and a January group that remained in the rearing system with progressively intensified restricted feeding while continuing to receive postbiotic supplementation until slaughter. This design allows for evaluation of postbiotic effects under two contrasting fattening protocols, as well as assessment of the methodological impact of 24 h carcass chilling on intestinal microbiome profiles.

The specific aims of this study were: (1) to characterize and compare ileal and caecal microbiota in geese (*A. anser*) supplemented with 2 g/kg Burain^®^ postbiotic or unsupplemented controls under two fattening protocols; (2) to evaluate the association between fattening protocol—force-feeding versus progressively intensified restricted feeding—on gut microbiome composition, noting from the outset that fattening protocol co-varies with age at slaughter, feeding intensity, and duration/continuity of postbiotic exposure between the two cohorts, and that the present design does not allow these factors to be experimentally separated; and (3) to assess whether 24 h post-slaughter chilling of carcasses prior to intestinal sampling alters the apparent microbial community composition compared to immediate warm sampling.

## 2. Materials and Methods

### 2.1. Ethic Statement

The experimental protocol was authorized by the Workplace Animal Welfare Committee of Hungarian University of Agriculture and Life Sciences (MATE-MKK-2020/22) because this work was not covered by the 40/2013 (II. 14) government decree on animal experiments.

### 2.2. Birds and Experimental Design

A total of 240 one-day-old (mixed sex) goslings (INTEGRAL MB 09 hybrid, Kisbéri Lúdtenyésztő Kft., Gödöllő, Hungary) were obtained from a local hatchery (AB OVO Kft., Újlengyel, Hungary) and allocated into two equal groups: an unsupplemented control group and a postbiotic-supplemented group (2 g/kg Burain^®^), each consisting of 120 birds subdivided into six replicates of 20 animals. The dry form of Burain^®^ (Kardamom Pharma Kft., Tiszabura, Hungary; EU Reg. No. 10 1 00307) contained 500 g lysate per kg: 300 g *Lactobacillus reuteri* SG L 01, 100 g *Lactobacillus acidophilus* SG L 11, and 100 g *Bifidobacterium longum* SGB 05, with 250 g wholegrain oatmeal and 250 g oat bran as substrate. The bacterial component was subjected to tyndallisation-based heat inactivation during production, resulting in non-viable microbial material. Vaccination against *Riemerella anatipestifer* and *Salmonella enterica* was administered subcutaneously at 10 days of age. All the geese were kept in chopped straw deep litter; 20 animals were kept in one unit (2 × 3.15 m). For the first 4 days, the number of light hours was 24 h, then it decreases gradually, by 1 h per day, to 17 h until the end of the period. Granulated feed and drinking water were provided *ad libitum* throughout the rearing phase. Birds were fed starter and grower diets at ages 1–21 and 22–56 days, respectively (Monortáp Kft., Csévharaszt, Hungary; composition presented in Table 1). Postbiotic supplementation at 2 g/kg was maintained continuously throughout both the starter and grower phases in the supplemented group.

At the end of the eight-week rearing period (day 56), the two groups were each divided into two subgroups, resulting in two experimental and three control groups in total (G1–G5, as detailed below). One subgroup from each treatment was transferred to individual force-feeding cages and subjected to a 14-day force-feeding protocol (December cohort; groups G1 and G2), while the remaining birds continued in the original housing under a restricted feeding protocol for a further six weeks (January cohort; groups G4 and G5). An additional subgroup from the control animals of the December cohort was used to assess the effect of post-mortem chilling on microbiome composition (group G3). Postbiotic supplementation was discontinued for force-feeding group from day 56 onwards, but continued for the restricted feeding group.

Following the eight-week rearing period, each of the four fattening-protocol subgroups (G1, G2, G4, G5) comprised 60 birds, distributed across six replicate pens of ten birds per subgroup. G3 was not a separate treatment subgroup but consisted of additional birds drawn from the same G1 (un-supplemented, force-fed) population, sampled via chilled (24 h) rather than warm dissection to assess the effect of post-mortem chilling; G1 and G3 therefore originated from the same treatment population but represent different individual birds. As immediate post-slaughter (warm) dissection is not part of the standard slaughterhouse protocol, only a limited number of birds per subgroup could be intercepted for warm intestinal sampling. Within each subgroup, animals selected for microbiome analysis were chosen at random, with selection performed independently within each replicate pen to ensure representation across the original experimental replicates. No a priori sample size or power calculation was performed for the microbiome analysis; the number of animals per group (n = 3–4) reflects the practical constraints of intestinal content collection during commercial slaughter, as detailed above.

During the force-feeding period (groups G1 and G2), birds received a maize-based diet (28% grain maize, 70% maize semolina, 2% premix) without postbiotic supplementation. Feed was provided in two daily meals initially, increasing to four meals by the end of the 14-day period, with individual portions increasing from 100 g to 400 g per meal.

During the restricted feeding period (groups G4 and G5), birds remained in the original group housing and received a commercial complete feed (Bonafarm-Bábolna Takarmány Kft., Nagyigmánd, Hungary; composition presented in Table 2) with postbiotic supplementation. Feed was provided in increasing meal frequency and portion size over the six-week period: two meals per day of 1350 g in weeks 1–2, three meals per day of 1100 g in week 3, three meals per day of 1300 g in week 4, and four meals per day of 1050–1150 g in weeks 5–6.

The December cohort (G1, G2, G3) was slaughtered at a commercial slaughterhouse (Integrál Zrt., Kiskunfélegyháza, Hungary) on 11 December 2024, following a 193 km transport initiated before 06:00. From each group, intestinal contents were collected from four animals (n = 4 per group per intestinal segment) immediately after slaughter under warm carcass conditions (warm dissection; groups G1 and G2). To assess the effect of post-mortem chilling on apparent microbiome composition, an additional set of control animals (group G3) was dissected after 24 h of chilling at 4 °C (cold dissection; 12 December 2024).

The January cohort (G4 and G5) was slaughtered on 7 January 2025 at the same facility, following the same transport conditions. Intestinal samples were collected immediately after slaughter under warm carcass conditions (n = 4 per group per intestinal segment).

For all samples, ileal and caecal contents were collected separately under sterile conditions using sterile instruments and collection vessels but from the same animals. Samples were immediately stored on ice and transported to the laboratory under refrigerated conditions, where they were stored at −80 °C until DNA extraction. Two samples failed to yield sufficient DNA and were excluded from further analysis (G2_I_04 and G3_I_04), resulting in a final dataset of 38 samples.

**Figure 1:**
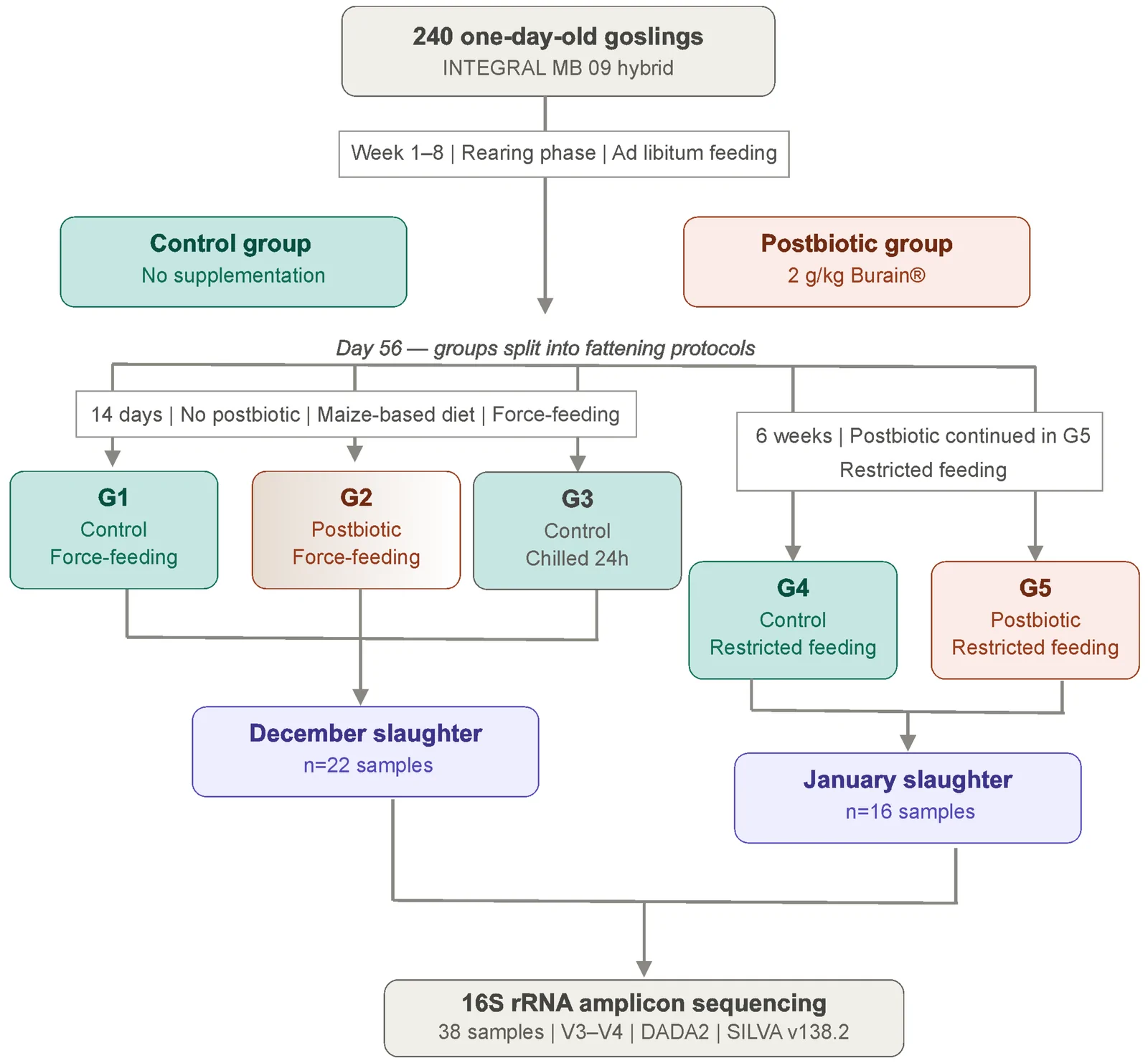
Flowchart of the experimental design. Goslings (n = 240) were reared for 8 weeks ± postbiotic (2 g/kg Burain^®^) and split into force-feeding (14 days) and restricted feeding (6 weeks) regimes. Intestinal samples were collected in December (n = 22; G1–G3) and January (n = 16; G4–G5) slaughters. Bacterial community structure was profiled by 16S rRNA gene (V3–V4) sequencing, DADA2, and SILVA v138.2 assignment.

The experimental groups and sample identifiers used throughout this study are summarised in Table 3 and experimental design is outlined in the flowchart (Figure 1).

### 2.3. Measured Parameters

Mortality was recorded daily. Live body weight was weekly assessed in both groups throughout the restricted feeding period (n = 60/groups) (Veit Electronics, BAT1, Moravany, Czech Republic). During the force-feeding period, live body weight was not recorded.

After carcass dissection, the thigh, breast, and liver were weighed (G4 = 59, G5 = 57). In the January cohort, liver weight was recorded as a proxy measure, as the restricted feeding protocol does not result in foie gras production; liver weight values are therefore not directly comparable to those of force-fed animals. The CIELAB system was utilised to evaluate liver colour parameters (lightness, L*; redness, a*; yellowness, b*). Measurements were taken on 20 samples from each group by Minolta R-400 Chromameter with 8 mm head (Konica Minolta Sensing, Inc., Osaka, Japan). The colour apparatus was calibrated against a white calibration plate before each reading.

The stiffness measurements were performed using a TA.XT Plus texture analyser (Stable Micro Systems, Godalming, Surrey, UK) equipped with spherical probe (n = 20/groups). The stiffness was calculated based on the force per unit time (g) diagram using Exponent Connect software. The measurements were carried on the right cranial end of the liver, on the ventral surface of the abdominal part. The spherical probe was inserted at a depth of 2 cm vertically into the specimen with three technical replicates. Statistical analyses of production parameters were performed using R software (R Core Team, 2013). Normality of data distribution was assessed using the Shapiro–Wilk test. In production parameters, each treatment comprised 60 geese housed in six pens, with 10 geese/pen. Each pen was considered an experimental replicate and served as the experimental unit for statistical analysis. Weekly live body weight measurements were analysed using a linear mixed-effects model, with supplementation, week, and the supplementation × week interaction as fixed effects and pen as a random effect. The significance of fixed effects was assessed using Type III tests with Satterthwaite’s approximation for the degrees of freedom. Estimated marginal means were used to compare supplementation group and control at each week, with Tukey adjustment applied for multiple comparisons. Group differences in carcass parameters and liver quality traits were evaluated by one-way analysis of variance (ANOVA) followed by Tukey’s honest significant difference (HSD) post hoc test for pairwise comparisons. Significance was accepted at *p* ≤ 0.05.

### 2.4. DNA Isolation and Amplicon Sequencing

Microbial community DNA was extracted from ileal and caecal contents using the RTP^®^ Pathogen Kit (Invitek Molecular, Berlin, Germany) following the manufacturer’s instructions, which includes a bead-beating step for mechanical cell lysis. The quality and integrity of extracted DNA were assessed by agarose gel electrophoresis and quantified using a NanoDrop One Microvolume UV-Vis spectrophotometer (Thermo Fisher Scientific, Waltham, MA, USA).

For taxonomic profiling, the variable V3–V4 region of the 16S rRNA gene was amplified with primers carrying Illumina adapter overhangs as described by Klindworth et al. [19]. Each PCR reaction contained 12.5 ng template DNA, 0.2 μM of each primer, and 12.5 μL of 2× Platinum™ II Hot-Start Green PCR Master Mix (Thermo Fisher Scientific), adjusted with Milli-Q water to a final volume of 25 μL. PCR cycling was performed in a ProFlex PCR System (Life Technologies, Carlsbad, CA, USA) with the following program: initial denaturation at 95 °C for 3 min; 25 cycles of 95 °C for 30 s, 55 °C for 30 s, and 72 °C for 30 s; followed by a final extension at 72 °C for 5 min. Amplicons were verified by agarose gel electrophoresis. Each PCR run included a no-template negative control, consisting of the identical reaction mixture with nuclease-free water substituted for template DNA. No amplification product was observed in any negative control by agarose gel electrophoresis, and negative controls were not carried forward into library preparation or sequencing. Sequencing libraries were prepared and subjected to paired-end sequencing on an Illumina MiSeq platform using the MiSeq Reagent Kit v2 (500 cycles) at Eurofins BIOMI Kft. (Gödöllő, Hungary). Primary base-calling was performed with bcl2fastq software (v2.17.1.14, Illumina). The amplicon sequencing data have been deposited in the NCBI Sequence Read Archive under BioProject accession number [PRJNA1506723].

### 2.5. Bioinformatics, Diversity and Statistical Analysis

Raw sequence reads were quality-processed prior to downstream analysis. PCR primers were trimmed using Cutadapt (v4.4) [20], allowing for no ambiguous nucleotides (“N”) in the input reads. Reads shorter than 50 nt after trimming were discarded. Read pre-processing and abundance analysis were performed with the DADA2 R package (v1.40.0) [21]. During filtering, no trimming based on nucleotide quality values was applied. Forward and reverse reads were retained in their full length, but sequences containing any “N” nucleotides were removed. Reads shorter than 100 nt were discarded. Maximum expected errors were set to 2 for both forward and reverse reads. Paired-end reads were merged if the overlap region was ≥12 nt with zero mismatches allowed. ASVs were retained if their merged length ranged from 350 to 500 nt, after prior removal of primer sequences with Cutadapt. Taxonomic assignment of merged ASVs was performed using the SILVA 16S rRNA gene reference database (v138.2), querying both orientations of merged reads and accepting assignments at a minimum bootstrap confidence of 75. The V3–V4 region has limited resolution below genus level for some taxa; species-level names reported here are the best-matching SILVA reference and should be read as indicative, not definitive—consistent with the genus-first phrasing used throughout the Results (e.g., “*Romboutsia*, specifically *R. ilealis*”). ASVs assigned to Chloroplast or Mitochondria were excluded from all downstream analyses. A total of 38 samples were processed through the pipeline. Raw read counts per sample ranged from 77,924 to 124,992 paired-end reads. Following primer trimming, quality filtering, denoising, merging, and chimera removal, between 34,310 and 66,071 non-chimeric reads were retained per sample. The DADA2 workflow identified 13,155 unique ASVs across all samples, of which 10,264 were identified as chimeric sequences and 14 failed to meet the length criteria (350–500 nt); these were excluded from further analysis, yielding a final dataset of 2877 bona fide ASVs. Rarefaction curves indicated that sequencing depth was sufficient to capture the microbial diversity in all samples, as curves reached saturation prior to the maximum sequencing depth.

Diversity analyses were performed to characterize microbial communities across samples. Alpha diversity metrics (Chao1, Shannon, and Simpson indices) were calculated to describe within-sample richness and evenness. Statistical differences in alpha diversity indices between the ten pre-specified group/segment comparisons were assessed on both rarefied and raw abundance data using the Conover–Iman test following a Kruskal–Wallis test, with Benjamini–Hochberg (BH) false discovery rate correction [22] across the ten comparisons; the Conover–Iman test [23] was adopted as the primary post hoc method, as it retains adequate statistical power for small, unequal group sizes (n = 3–4) compared with the more conservative Wilcoxon rank-sum [24] and Nemenyi tests [25], which are reported alongside it as sensitivity analyses (Supplementary Table S2). Beta diversity was assessed using Bray–Curtis dissimilarity matrices calculated from relative abundance data, and visualised by principal coordinates analysis (PCoA). To quantify the contributions of experimental factors to microbial community composition, permutational multivariate analysis of variance (PERMANOVA) was performed using the adonis2 function of the vegan R package (v2.6) [26] with 9999 permutations, applied to the same ten pairwise group comparisons; homogeneity of multivariate dispersion was assessed for each comparison using PERMDISP (betadisper, Bray–Curtis dissimilarity, 999 permutations), and Benjamini–Hochberg correction was additionally applied across the ten PERMANOVA comparisons. To identify taxa significantly associated with specific experimental groups, Linear Discriminant Analysis Effect Size (LEfSe) was implemented using the lefser R package [27], with pairwise comparisons performed between: G1 vs. G2 (postbiotic effect under force-feeding), G4 vs. G5 (postbiotic effect under restricted feeding), G1 vs. G4 (protocol related differences), and G1 vs. G3 (effect of post-mortem chilling). An LDA score threshold of 2.0 was applied, with underlying Kruskal–Wallis and pairwise Wilcoxon significance tests set at α = 0.05. Core microbiome analysis was performed to identify shared and unique taxa across experimental groups and intestinal locations. Taxa present in at least 80% of samples within a given group were defined as core members. Results were visualised using Venn diagrams for two-way comparisons (ileum vs. caecum) and UpSet plots for multi-group comparisons (G1–G5), generated with the ggVennDiagram R package [28].

All analyses were performed in R (version 4.4.2) using the RStudio integrated development environment (version 2025.05.0),whereas visualization of diversity indices and ordination plots was carried out using the ggplot2 package, with colour palettes applied from the paletteer library to improve graphical clarity and accessibility [29,30]. Detailed phylogenetic results of the DADA2 analysis are provided in Supplementary Table S1.

### 2.6. Declaration of Generative AI and AI-Assisted Technologies in the Writing Process

During the preparation of this work, the authors used Generative AI technologies, specifically ChatGPT (GPT-5.5, OpenAI), Claude (Sonnet 5, Anthropic), and Gemini (Flash, Google), solely for language editing, style improvement, grammar refinement, and assisting with R code formatting for data visualization. Following the use of these tools, the authors reviewed, edited, and verified all outputs and take full responsibility for the content, accuracy, and scientific integrity of the final publication.

## 3. Results

### 3.1. Production Performance Under Restricted Feeding (January Cohort)

Mortality was low in both groups, with one animal lost in the control group (G4) and three in the postbiotic-supplemented group (G5) during the six-week restricted feeding period. As expected, there was a significant main effect of week (F(5, 686.04) = 28.10, *p* < 0.001), reflecting normal growth over the study period. There was no significant main effect of supplementation (F(1, 10) = 0.56, *p* = 0.471) and no significant supplementation × week interaction (F(5, 686.04) = 0.47, *p* = 0.796). No significant differences in live body weight were detected between G4 and G5 at any of the weekly assessments from week 9 (G4 = 60, G5 = 60) through week 14 (G4 n = 59, G5 n = 57) (Tukey-adjusted *p* ≥ 0.142). (Table 4). Mean body weight at the final measurement (week 14) was 5936.1 ± 1086.0 g in G4 and 5889.5 ± 902.9 g in G5 (*p* > 0.05).

Values are presented as means ± standard deviation (SD), based on six pens per treatment, with 10 geese per pen. The pen was considered the experimental unit. Live body weight was analysed using a linear mixed-effects model with treatment, week, and treatment × week as fixed effects and pen as a random effect. a: Identical superscript letters indicate no significant differences between groups at the given week (Tukey-adjusted *p* ≥ 0.142). 

At slaughter, liver weight (G4 n = 59, G5 n = 57), stiffness, breast weight, and thigh weight (G4 and G5 n = 20/group) did not differ significantly between groups (Table 5). Liver colour parameters, however, showed significant differences: G4 (control) livers had significantly higher lightness (“L”: 43.5 ± 13.4 vs. 35.7 ± 3.2, *p* < 0.05) and yellowness (“b”: 15.6 ± 6.9 vs. 11.5 ± 2.5, *p* < 0.05) values compared to G5 (postbiotic-supplemented), while redness (“a”) did not differ between groups (12.6 ± 2.4 vs. 12.5 ± 1.9, *p* > 0.05). These results indicate that prior postbiotic supplementation during the rearing phase was associated with darker and less yellow liver colour at slaughter, without affecting liver weight or texture.

### 3.2. Phylum-Level Community Composition Reveals Intestinal Segment-Specific Patterns

**Figure 2:**
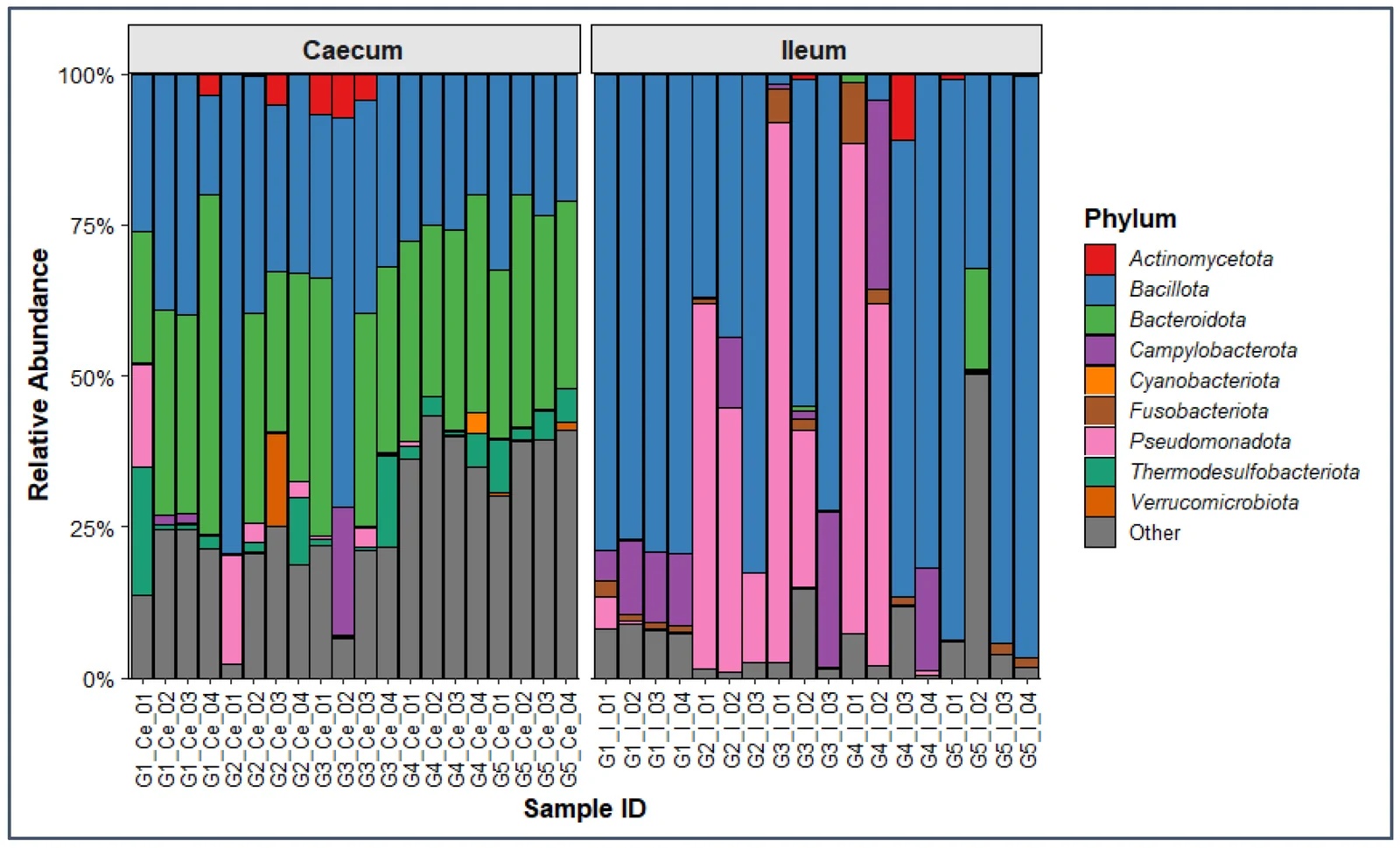
Relative abundance of the dominant bacterial phyla in individual ileal (I) and caecal (C) microbiome samples from geese in the five experimental groups (G1–G5). Each stacked bar represents one biological replicate, and colours indicate the relative abundance of individual bacterial phyla. Only phyla with a mean relative abundance ≥1% in at least one experimental group are shown.

Across all 38 samples, eight bacterial phyla were detected at mean relative abundances above 0.01% (Figure 2). The community was dominated by Bacillota and Bacteroidota, with Pseudomonadota, Campylobacterota, and Thermodesulfobacteriota present as secondary constituents. Fusobacteriota, Actinomycetota, and Verrucomicrobiota were detected at low mean abundances across most groups. Cyanobacteriota was present only in trace amounts in a subset of G4 and G5 caecal samples and is not discussed further.

The most prominent feature of the phylum-level composition was a strong spatial partitioning between the ileum and caecum. Bacillota was the dominant phylum in ileal samples across all groups (mean 60.2% ± 32.6), whereas its relative abundance was markedly lower in the caecum (mean 32.7% ± 15.2). In contrast, Bacteroidota was nearly absent from the ileum (mean 1.1% ± 3.9) but constituted a major component of the caecal microbiota across all groups (mean 30.5% ± 12.5). Thermodesulfobacteriota was exclusively detected in caecal samples (mean 4.3% ± 5.7 in caecum vs. 0.0% ± 0.1 in ileum), as was Verrucomicrobiota (mean 0.9% ± 3.4 in caecum). Fusobacteriota showed the opposite pattern, being present in ileal samples (mean 1.8% ± 2.5) but virtually absent from the caecum (mean 0.0% ± 0.0). Pseudomonadota and Campylobacterota showed high inter-individual variability in the ileum, with mean values of 21.3% ± 31.2 and 7.2% ± 9.7, respectively, reflecting large differences between individual samples within groups. Their caecal abundances were consistently low across all groups (Pseudomonadota: 2.3% ± 5.3; Campylobacterota: 1.3% ± 4.8). At the group level, Bacillota abundance in the ileum ranged from a mean of 40.4% ± 44.2 in G4 to 78.9% ± 31.2 in G5, with G1 showing the most consistent values across individuals (78.6% ± 1.0). Caecal Bacillota was notably lower and more consistent, ranging from 24.2% ± 5.7 (G5) to 44.9% ± 23.6 (G2). Bacteroidota in the caecum was relatively consistent across groups, ranging from 24.0% ± 16.4 (G2) to 36.3% ± 14.4 (G1), with all January groups (G4: 32.7% ± 3.2; G5: 32.4% ± 4.4) showing lower inter-individual variability than December groups. Pseudomonadota showed a notable contrast between December and January ileal samples: G2 (39.6% ± 23.2), G3 (38.5% ± 45.9), and G4 (35.4% ± 41.5) all showed high mean values with extreme variability driven by individual samples, whereas G1 (1.6% ± 2.6) and G5 (0.1% ± 0.0) were substantially lower. Thermodesulfobacteriota was present in all caecal groups with means ranging from 2.8% ± 2.0 (G4) to 6.2% ± 9.9 (G1), with high variability reflecting its presence in only a subset of individuals within each group.

### 3.3. Genus-Level Community Composition Across Experimental Comparisons

Genus-level relative abundances presented in this section are descriptive summaries and were not independently statistically tested for each taxon; apparent differences—particularly where driven by one or two individual animals, as noted below—should therefore be interpreted as illustrative of compositional patterns rather than as confirmed statistical effects. Taxon-level differences supported by formal statistical testing are reported separately via LEfSe (Section 3.3.5).

#### 3.3.1. Effect of Fattening Protocol on Genus-Level Composition: Force-Feeding Versus Restricted Feeding (G1 vs. G4) (Figure 3)

##### Ileum

The ileal microbiota differed markedly between the force-fed December control group (G1) and the restricted-fed January control group (G4), with high inter-individual variability observed within both groups. In G1, the community was dominated by *Romboutsia* (specifically *Romboutsia ilealis*) (mean 44.83% ± 31.83), which was virtually absent in G4 (0.19% ± 0.12). *Clostridium* (11.08% ± 12.76) and *Enterococcus* (12.53% ± 24.13) were also prominent in G1 but nearly undetectable in G4 (0.18% ± 0.36 and 1.06% ± 2.09, respectively). *Campylobacter* (identified as *Campylobacter jejuni)* (3.69% ± 1.45) and *Helicobacter* (6.51% ± 4.53) were present in G1, with *Helicobacter* also detected at notable levels in G4 (12.07% ± 15.08), though with high variability. *Lactobacillus* (identified predominantly as *Lactobacillus johnsonii)* was detected in G1 (2.61% ± 4.83) but was absent from G4. In contrast, G4 was characterised by high relative abundance of *Escherichia–Shigella* (represented by *Escherichia coli*) (34.86% ± 40.61) and *Ligilactobacillus* (32.32% ± 37.20), both of which showed extreme inter-individual variation. *Cetobacterium* was present in both groups but at higher mean abundance in G4 (3.56% ± 4.57) than in G1 (1.53% ± 0.61).

##### Caecum

The caecal microbiota showed a distinct genus-level composition between G1 and G4, with less inter-individual variability than the ileum, particularly in G4. *Bacteroides* was the dominant genus in both groups, present at higher mean abundance in G4 (24.73% ± 2.73) than in G1 (17.29% ± 5.72). *Christensenellaceae* unclassified was exclusively detected in G4 (6.77% ± 1.65) and was completely absent from G1. *Oscillospira* (2.48% ± 1.29) and *Oscillospiraceae* UCG-005 (1.39% ± 0.43) were also more abundant in G4, while *Desulfovibrio* was higher in G4 (2.57% ± 2.09) compared to G1 (0.56% ± 0.19). *Veillonellaceae* unclassified showed higher abundance in G4 (1.57% ± 0.07) than in G1 (0.68% ± 0.46). Conversely, several taxa were more abundant in G1 than in G4. *Prevotellaceae* unclassified showed markedly higher mean abundance in G1 (8.69% ± 8.88) compared to G4 (0.41% ± 0.10), although with high variability. *Alistipes* (8.07% ± 8.76 vs. 2.28% ± 1.52), *Rikenellaceae* RC9 gut group (5.78% ± 5.43 vs. 1.07% ± 0.53), *Oscillospirales* unclassified (4.32% ± 1.38 vs. 1.92% ± 0.21), and *Faecalibacterium* (2.48% ± 1.60 vs. 0.37% ± 0.14) were all higher in G1. *Bilophila* (identified as *Bilophila wadsworthia*) (5.29% ± 10.29) and *Escherichia–Shigella* (represented by *Escherichia coli*) (4.32% ± 8.52) were detected in G1 at variable levels but were nearly absent from G4.

**Figure 3:**
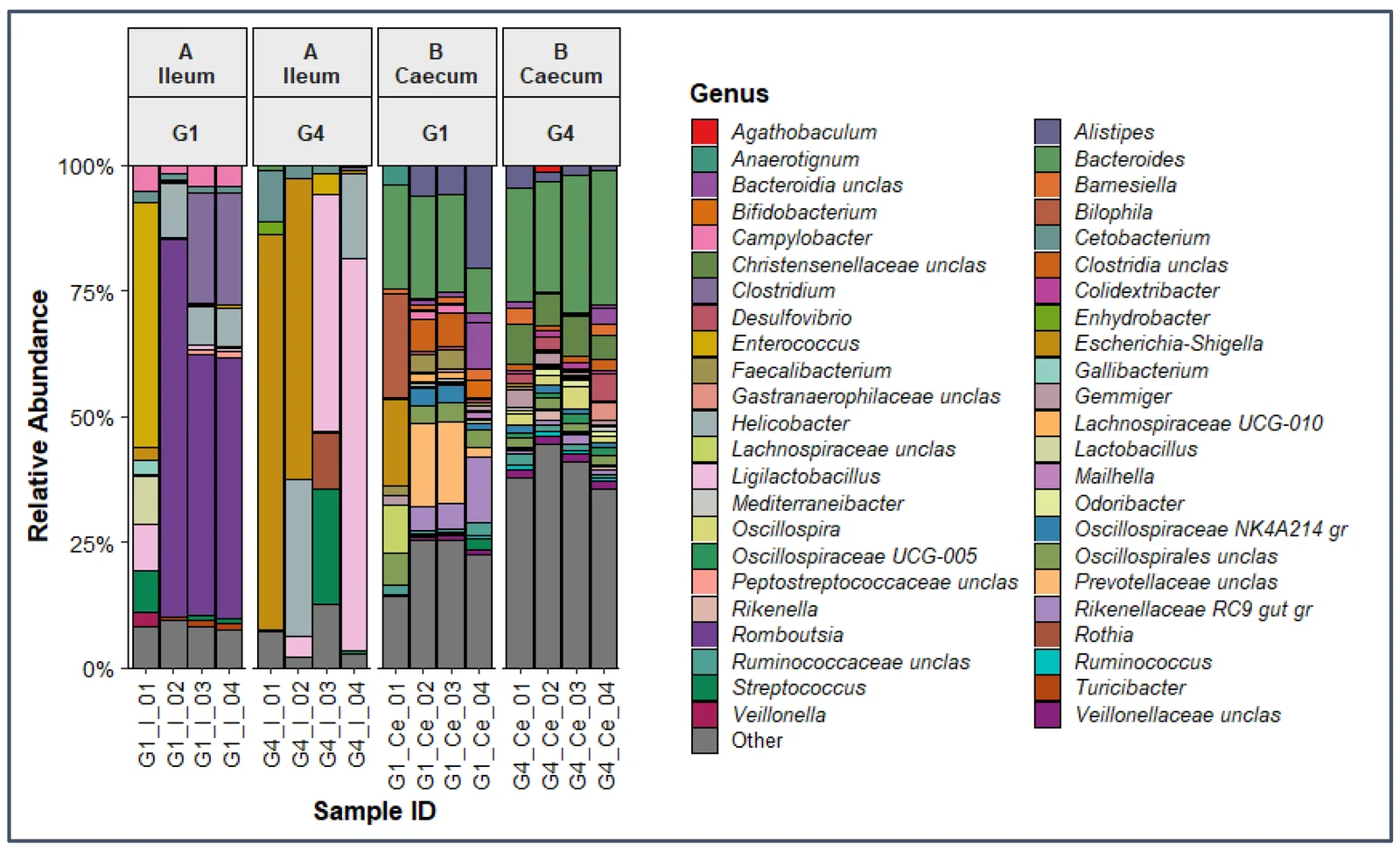
Relative abundance of the dominant bacterial genera in ileal (A) and caecal (B) microbiome samples from force-fed (G1) and restricted-fed control (G4) geese. Each stacked bar represents one biological replicate, and colours indicate the relative abundance of individual bacterial genera. Only genera with a mean relative abundance ≥1% in at least one experimental group are shown.

#### 3.3.2. Effect of Postbiotic Supplementation Under Force-Feeding Conditions (G1 vs. G2) (Figure 4)

##### Ileum

The ileal microbiota showed marked compositional differences between the unsupplemented force-fed control group (G1) and the postbiotic-supplemented force-fed group (G2), with high inter-individual variability in both groups. G1 was dominated by *Romboutsia* (specifically *Romboutsia ilealis*) (44.83% ± 31.83), which was virtually absent from G2 (0.01% ± 0.02). *Clostridium* (11.08% ± 12.76) and *Enterococcus* (12.53% ± 24.13) were prominent in G1 but nearly undetectable in G2 (0.39% ± 0.65 and 0.38% ± 0.44, respectively). *Helicobacter* was present at notable abundance in G1 (6.51% ± 4.53) but essentially absent from G2 (0.08% ± 0.12). *Cetobacterium* was detected in G1 (1.53% ± 0.61) but at low abundance in G2 (0.28% ± 0.47). In contrast, G2 was characterised by high relative abundance of *Escherichia–Shigella* (represented by *Escherichia coli*) (39.62% ± 23.21), *Lactobacillus* (29.01% ± 34.95), and *Ligilactobacillus* (19.64% ± 16.65), all showing extreme inter-individual variability. *Streptococcus* (driven by *Streptococcus gallolyticus*) was also elevated in G2 (4.64% ± 6.86) compared to G1 (2.55% ± 3.72). *Campylobacter* (represented by *Campylobacter jejuni*) was detected in both groups at similar mean abundance (G1: 3.69% ± 1.45; G2: 3.85% ± 6.67), though with higher variability in G2.

##### Caecum

The caecal microbiota also differed between G1 and G2, though with partially overlapping compositions. *Bacteroides* was the dominant genus in both groups but showed notably higher abundance in G1 (17.29% ± 5.72) compared to G2 (9.21% ± 6.86). *Prevotellaceae* unclassified was markedly higher in G1 (8.69% ± 8.88) than in G2 (0.69% ± 0.69), as were *Alistipes* (8.07% ± 8.76 vs. 8.20% ± 8.51—nearly identical), *Faecalibacterium* (2.48% ± 1.60 vs. 0.85% ± 1.00), and *Bilophila* (5.29% ± 10.29 vs. 2.77% ± 5.54). G2 showed elevated *Streptococcus* (7.37% ± 6.01 vs. 0.68% ± 1.14 in G1), *Lactobacillus* (15.99% ± 30.84 vs. 0.14% ± 0.26), *Ruminococcaceae* unclassified (3.94% ± 3.53 vs. 1.41% ± 0.98), and *Akkermansia* (3.81% ± 7.58 vs. 0.01% ± 0.02). *Megamonas* (specifically *Megamonas hypermegale*) was detected exclusively in G2 at low but notable mean abundance (1.04% ± 1.58) and was virtually absent from G1 (0.05% ± 0.07). *Gemmiger* was also higher in G2 (1.72% ± 3.07) than in G1 (0.92% ± 0.77). *Escherichia–Shigella* (represented by *Escherichia coli*) and *Bilophila* (identified as *Bilophila wadsworthia*) were present in both groups with high variability, driven by extreme values in individual samples.

**Figure 4:**
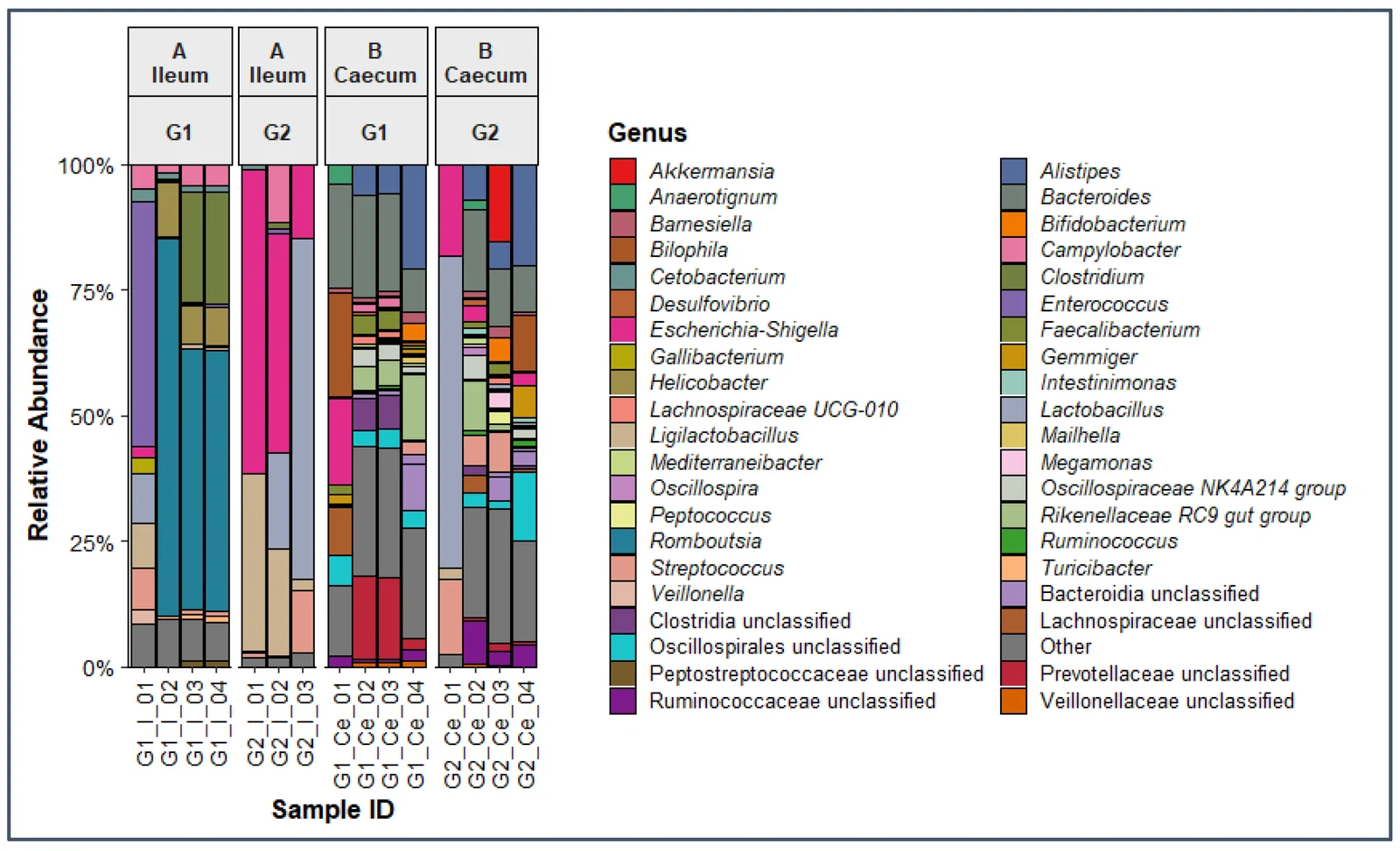
Relative abundance of the dominant bacterial genera in ileal (A) and caecal (B) microbiome samples from force-fed control (G1) and force-fed postbiotic-supplemented (G2) geese. Each stacked bar represents one biological replicate, and colours indicate the relative abundance of individual bacterial genera. Only genera with a mean relative abundance ≥1% in at least one experimental group are shown.

#### 3.3.3. Effect of Postbiotic Supplementation Under Restricted Feeding Conditions (G4 vs. G5) (Figure 5)

##### Ileum

The ileal microbiota of the restricted-fed control group (G4) and the postbiotic-supplemented restricted-fed group (G5) both showed high inter-individual variability, with *Ligilactobacillus* as the dominant genus in both groups. Mean abundance of *Ligilactobacillus* was 32.32% ± 37.20 in G4 and 54.36% ± 40.31 in G5, reflecting the large intra-group variation driven by individual samples. The most pronounced difference between the groups was the near-complete absence of *Escherichia–Shigella* (represented by *Escherichia coli*) in G5 (0.05% ± 0.03) compared to its high but variable abundance in G4 (34.86% ± 40.61), where two individuals showed dominance of this genus while two others did not. Similarly, *Helicobacter* was detected at notable mean abundance in G4 (12.07% ± 15.08) but was essentially absent from G5 (0.01% ± 0.03). *Cetobacterium* was higher in G4 (3.56% ± 4.57) than in G5 (0.83% ± 0.95).

**Figure 5:**
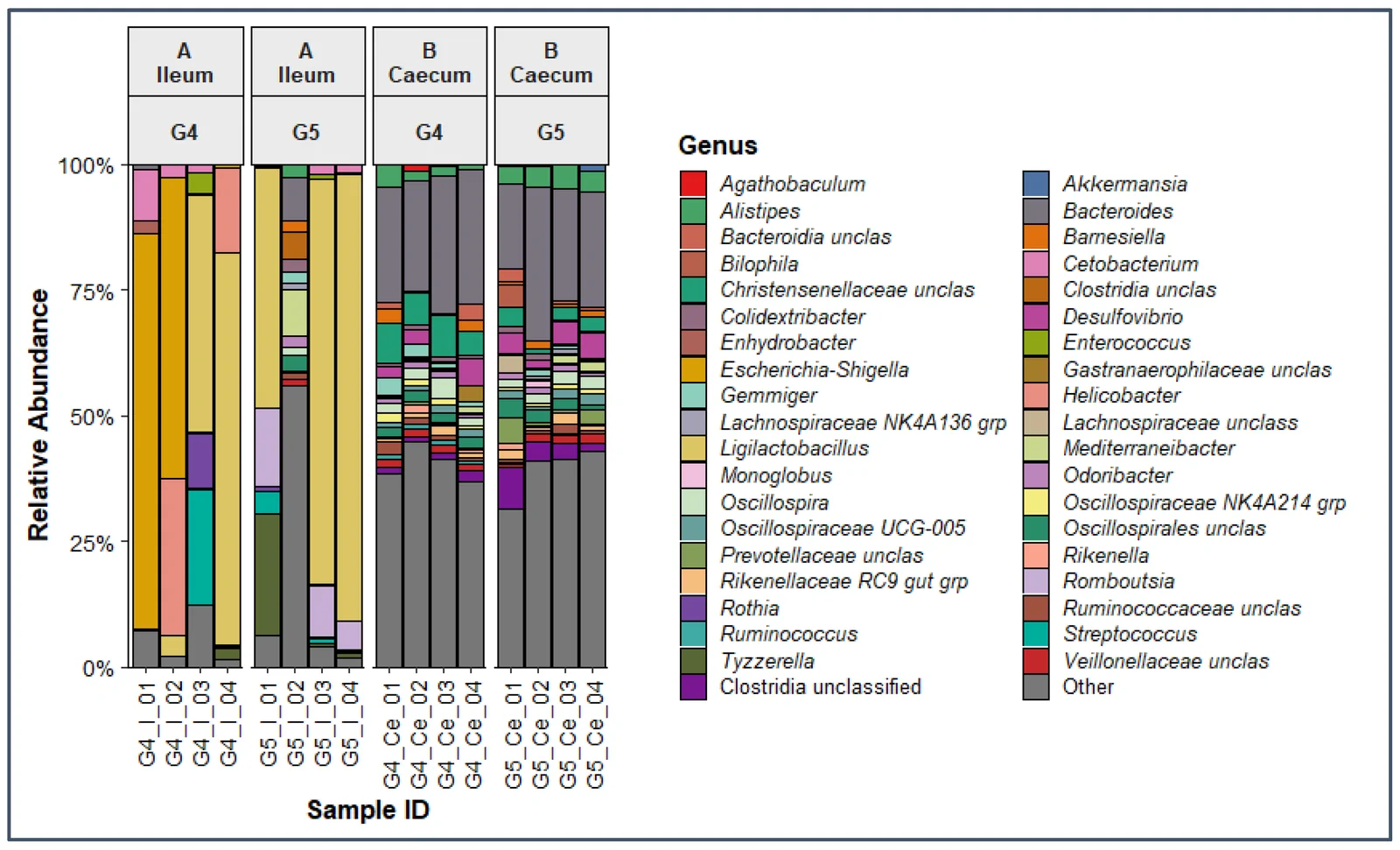
Relative abundance of the dominant bacterial genera in ileal (A) and caecal (B) microbiome samples from restricted-fed control (G4) and restricted-fed postbiotic-supplemented (G5) geese. Each stacked bar represents one biological replicate, and colours indicate the relative abundance of individual bacterial genera. Only genera with a mean relative abundance ≥1% in at least one experimental group are shown.

Conversely, several taxa were elevated in G5 compared to G4. *Romboutsia* (specifically *Romboutsia ilealis*) was detected at 7.92% ± 6.64 in G5 but was virtually absent from G4 (0.19% ± 0.12). *Tyzzerella* was present at 6.43% ± 11.97 in G5 compared to 0.61% ± 1.09 in G4, though with extreme variability driven by a single individual. *Mediterraneibacter* (2.36% ± 4.67) and *Clostridia* unclassified (1.31% ± 2.58) were also detected in G5 at low but notable mean abundances, while being essentially absent from G4. *Streptococcus* (including *Streptococcus suis*) showed higher mean abundance in G4 (5.83% ± 11.41) than in G5 (1.49% ± 1.99), though both groups showed high variability.

##### Caecum

The caecal microbiota showed more consistent patterns between G4 and G5 than the ileum, with *Bacteroides* (characterized by *Bacteroides dorei*, *Bacteroides massiliensis and Bacteroides vulgatus*) dominating both groups at nearly identical mean abundances (G4: 24.73% ± 2.73; G5: 23.20% ± 5.54). *Christensenellaceae* unclassified was markedly higher in G4 (6.77% ± 1.65) than in G5 (2.48% ± 1.22), representing one of the most consistent inter-group differences in the caecum. *Gemmiger* was also higher in G4 (1.91% ± 1.27) compared to G5 (0.66% ± 0.60), as were *Ruminococcaceae* unclassified (1.37% ± 0.70 vs. 0.89% ± 0.61) and *Barnesiella* (represented by *Barnesiella viscericola*) (1.46% ± 1.41 vs. 0.98% ± 0.51). In contrast, *Clostridia* unclassified was higher in G5 (4.22% ± 2.79) than in G4 (1.44% ± 0.50). *Alistipes* (predominantly *Alistipes inops*) was elevated in G5 (4.09% ± 0.56) compared to G4 (2.28% ± 1.52), with notably lower variability in G5. *Prevotellaceae* unclassified showed higher mean abundance in G5 (2.23% ± 2.06) than in G4 (0.41% ± 0.10), though with high inter-individual variability in G5. *Desulfovibrio* (represented by *Desulfovibrio piger*) was also higher in G5 (3.91% ± 1.58) than in G4 (2.57% ± 2.09). Multiple *Oscillospiraceae*-affiliated taxa (*Oscillospira*, *Oscillospiraceae* UCG-005, *Oscillospirales* unclassified) were present at comparable abundances in both groups, suggesting these are stable community members irrespective of postbiotic supplementation under restricted feeding conditions.

#### 3.3.4. Effect of Post-Mortem Chilling on Genus-Level Composition (G1 vs. G3) (Figure 6)

##### Ileum

The ileal microbiota showed marked compositional differences between the warm-dissected (G1) and chilled (G3) control groups, despite originating from the same cohort of force-fed animals. G1 was dominated by *Romboutsia* (specifically *Romboutsia ilealis*) (44.83% ± 31.83), which was virtually absent from G3 (0.02% ± 0.03). *Helicobacter* was prominent in G1 (6.51% ± 4.53) but essentially undetectable in G3 (0.01% ± 0.02). *Cetobacterium* was present in G1 (1.53% ± 0.61) but nearly absent from G3 (0.08% ± 0.10). *Clostridium* (11.08% ± 12.76) and *Enterococcus* (represented by *Enterococcus cecorum*) (12.53% ± 24.13) were detected in G1 at variable abundances but were substantially lower in G3 (4.59% ± 7.94 and 3.58% ± 4.87, respectively). In contrast, G3 showed markedly higher abundances of several taxa. *Escherichia–Shigella* (represented by *Escherichia coli*) dominated one individual in G3 at 89.43%, yielding a high but variable group mean (29.98% ± 51.49) compared to G1 (0.80% ± 1.08). *Lactobacillus* (identified predominantly as *Lactobacillus johnsonii*) (15.89% ± 16.51) and *Ligilactobacillus* (14.92% ± 18.19) were substantially elevated in G3 compared to G1 (2.61% ± 4.83 and 2.62% ± 4.24, respectively). *Campylobacter* showed higher mean abundance in G3 (9.40% ± 14.20) than in G1 (3.69% ± 1.45), driven by one individual with 25.79%. *Gallibacterium* (represented by *Gallibacterium anatis*) was detected at notable mean abundance in G3 (8.54% ± 14.79) but was nearly absent from G1 (0.77% ± 1.54), again driven by a single individual (25.62%). *Fusobacterium* identified as *Fusobacterium mortiferum*) was detected exclusively in G3 (2.44% ± 2.76) and was absent from G1.

**Figure 6:**
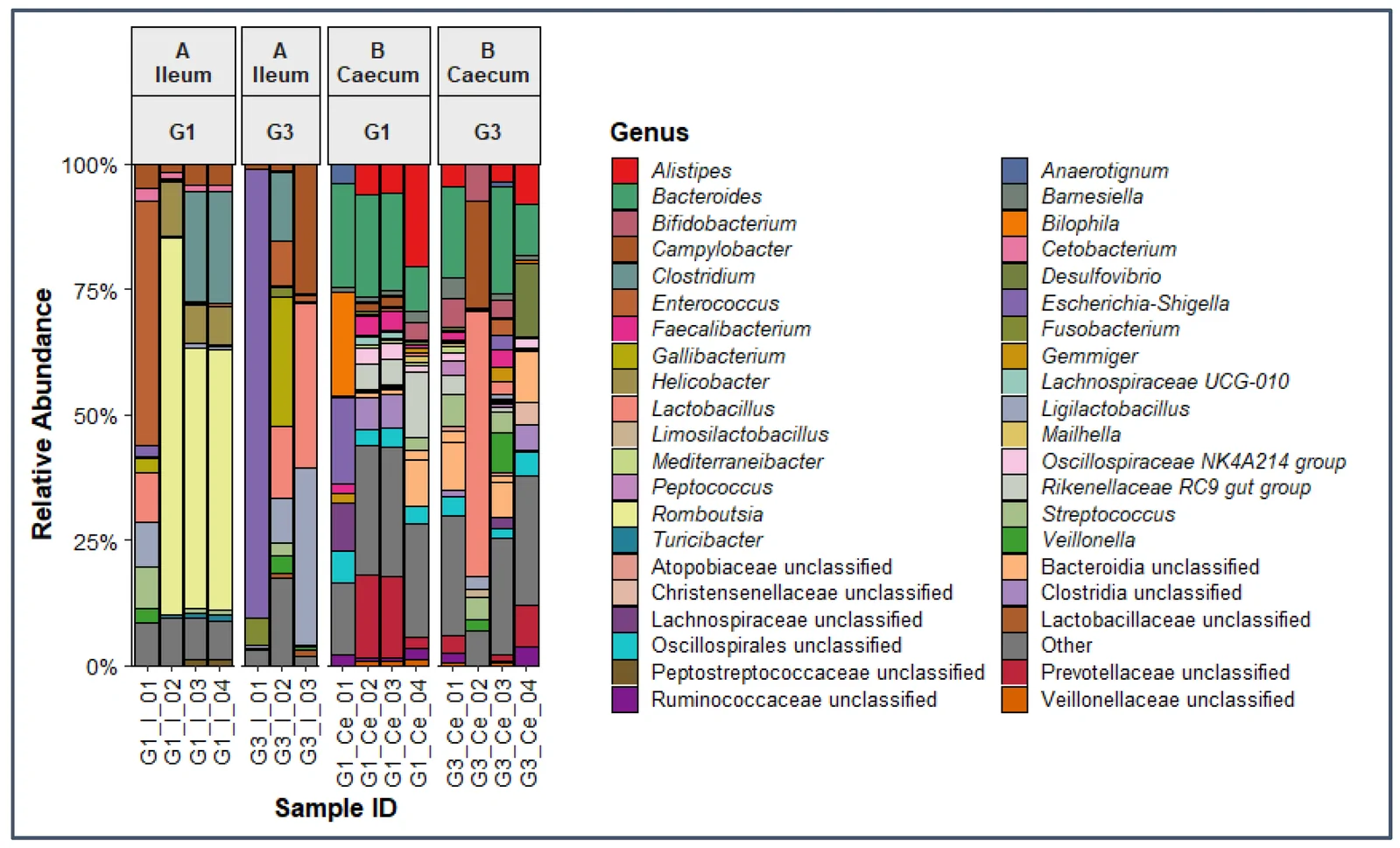
Relative abundance of the dominant bacterial genera in ileal (A) and caecal (B) microbiome samples from warm-dissected (G1) and post-mortem chilled (G3) geese. Each stacked bar represents one biological replicate, and colours indicate the relative abundance of individual bacterial genera. Only genera with a mean relative abundance ≥1% in at least one experimental group are shown.

##### Caecum

The caecal microbiota also differed between G1 and G3, though the differences were generally less pronounced than in the ileum. *Bacteroides* remained the dominant genus in both groups but at lower mean abundance in G3 (12.39% ± 9.48) compared to G1 (17.29% ± 5.72), with higher variability in G3 driven by one individual with no detectable *Bacteroides*. *Bifidobacterium* (containing *Bifidobacterium pseudolongum* and *Bifidobacterium pullorum*) was markedly higher in G3 (4.17% ± 3.13) than in G1 (0.95% ± 1.73). *Lactobacillus* (predominantly *Lactobacillus johnsonii*) was substantially elevated in G3 (13.84% ± 26.01) compared to G1 (0.14% ± 0.26), though with extreme variability driven by one individual (52.81%). *Streptococcus* (including *Streptococcus gallolyticus*, *Streptococcus suis* and *Streptococcus moroccensis*) (3.81% ± 2.65 vs. 0.68% ± 1.14), *Veillonella* (identified as *Veillonella magna)* (2.57% ± 3.76 vs. 0.21% ± 0.22), *Campylobacter* (represented by *Campylobacter jejuni*) (5.44% ± 10.67 vs. 0.85% ± 0.89), *Desulfovibrio* (represented by *Desulfovibrio piger*) (3.81% ± 7.19 vs. 0.56% ± 0.19), and *Bacteroidia* unclassified (6.71% ± 4.73 vs. 2.86% ± 4.38) were all higher in G3. Conversely, *Prevotellaceae* unclassified (8.69% ± 8.88 vs. 3.34% ± 3.74), *Alistipes* (containing *Alistipes inops* and *Alistipes obesi*) (8.08% ± 8.76 vs. 3.97% ± 3.28), *Rikenellaceae* RC9 gut group (5.78% ± 5.43 vs. 1.13% ± 1.71), *Oscillospirales* unclassified (4.32% ± 1.38 vs. 2.55% ± 2.03), and *Bilophila* (identified as *Bilophila wadsworthia*) (5.29% ± 10.29 vs. 0.12% ± 0.24) were higher in G1 than in G3.

#### 3.3.5. Differential Abundance Analysis (LEfSe)

LEfSe (LDA threshold = 2.0; Kruskal–Wallis and pairwise Wilcoxon α = 0.05) was applied to identify the individual taxa driving the group-level differences described above. As LEfSe does not itself correct for multiple comparisons across taxa, and group sizes were small (n = 3–4), individual taxon-level findings should be interpreted with appropriate caution, particularly where not corroborated by the community-level (PERMANOVA) or descriptive relative-abundance results reported elsewhere in this study. Full ranked taxon lists for all ten pairwise comparisons, together with genus- and species-level assignments, are provided in Supplementary Figure S1; the two comparisons yielding the largest number of discriminant taxa (G1 vs. G4 and G2 vs. G5, caecum) are summarised below by their top 20 taxa per side (ranked by LDA score).

##### Fattening Protocol (G1 vs. G4)

Ileum. G4 was discriminated by six ASVs (LDA 2.1–4.2), all assigned to environmental or skin-associated taxa rather than typical gut commensals: *Ligilactobacillus* sp., *Priestia* sp., *Cutibacterium acnes*, *Acinetobacter johnsonii*, *Glycomyces* sp., and *Chitinophaga japonensis*. G1 was characterised by 21 discriminant ASVs (LDA −2.6 to −4.2): *Campylobacter jejuni* (two ASVs), *Lactobacillus johnsonii*, *Veillonella magna*, eight further ASVs assigned to *Romboutsia* (five of which resolved to *Romboutsia ilealis*), *Ligilactobacillus* sp., and *Streptococcus* sp.

Caecum. This comparison yielded by far the largest number of discriminant taxa. Among the top 20 for G1: four *Bacteroides* ASVs (LDA −3.5 to −4.5), *Bilophila* sp. (−4.35), two *Rikenellaceae* RC9 gut group ASVs, *Faecalibacterium* (two ASVs), an unclassified *Oscillospiraceae* member (the single strongest taxon in this comparison, −4.01), *Streptococcus gallolyticus*, *Sellimonas* sp., *Campylobacter jejuni*, an unclassified Clostridia vadinBB60 group member, and *Barnesiella* (two ASVs). Among the top 20 for G4: three *Bacteroides* ASVs (LDA 4.26–4.74, the strongest effects in this comparison), an unclassified *Christensenellaceae* member (4.51), *Alistipes* (two ASVs), *Rikenella*, an unclassified Rikenellaceae RC9 gut group member, *Odoribacter*, *Gemmiger*, *Colidextribacter*, *Ruminococcus*, *Bacteroides dorei*, two further ASVs resolved to *Bacteroides massiliensis*, and several unclassified Clostridia UCG-014/Oscillospiraceae/Veillonellaceae members. The scale and consistency of this signal mirrors the strong separation between December and January cohorts in the beta-diversity ordination (Section 3.5), consistent with cohort- and protocol-related differences shaping caecal community composition.

##### Postbiotic Effect Under Force-Feeding (G1 vs. G2)

Ileum. G2 was discriminated by a single ASV assigned to *Campylobacter jejuni* (LDA = 4.62). G1 was discriminated by a second *C. jejuni* ASV, two *Cetobacterium* ASVs, and an unclassified *Saccharimonadales* member (LDA −2.9 to −4.2).

Caecum. G2 was discriminated by *Streptococcus gallolyticus* (4.16) and *Butyricimonas* sp. (2.34). G1 was discriminated by an unclassified *Oscillospiraceae* member (the strongest effect, −3.95), *Oscillospira* sp., two *Desulfovibrio* ASVs, *Barnesiella*, *Ruminiclostridium*, and *Bacteroides*.

##### Postbiotic Effect Under Restricted Feeding (G4 vs. G5)

Ileum. G5 was discriminated by three *Romboutsia* ASVs, an unclassified *Christensenellaceae* member, and an unclassified Clostridia vadinBB60 group member (LDA 2.2–3.2). G4 was discriminated by *Escherichia coli* (the strongest effect, −5.13), *Cutibacterium acnes*, and an unclassified *Pseudonocardiaceae* member.

Caecum. G5 was discriminated by *Bacteroides dorei* (the strongest effect, 3.75), two *Lachnospiraceae* NK4A136 group ASVs, an unclassified Oscillospiraceae member, *Desulfovibrio*, an unclassified RF39-order member, *Christensenellaceae*, Clostridia UCG-014, *Intestinimonas* (two ASVs), *Lachnospiraceae* (two ASVs), *Alistipes* (two ASVs), and *Akkermansia*. G4 was discriminated by an unclassified *Christensenellaceae* member (the strongest effect, −4.37), *Ruminococcus*, two Clostridia UCG-014 ASVs, NK4A214 group, *Shuttleworthia*, *Alistipes*, *Acutalibacter*, two *Lachnospiraceae* ASVs, *Pygmaiobacter*, *Massiliomicrobiota*, *Christensenellaceae* R-7 group (two ASVs), and *Colidextribacter*.

##### Postbiotic Effect Across Both Protocols (G2 vs. G5)

Ileum. G5 was discriminated by *Turicibacter* and *Ligilactobacillus* (LDA 2.3–2.7). G2 was discriminated by *Escherichia coli* (the largest effect size observed across all comparisons, −5.11), *Lactobacillus johnsonii*, and *Campylobacter jejuni*.

Caecum. All 15 discriminant taxa for G2 are reported in full (no truncation needed): *Campylobacter jejuni* (the strongest effect, −4.40), *Bacteroides* (three ASVs), *Streptococcus gallolyticus*, *Bifidobacterium pseudolongum*, *Enterococcus cecorum*, *Ligilactobacillus*, *Alistipes*, *Desulfovibrio piger*, *Streptococcus* sp., *Peptococcus*, and *Intestinimonas*. Among the top 20 (of 48) for G5: *Bacteroides* (three ASVs, LDA 4.20–4.68, the strongest effects), *Christensenellaceae*, *Alistipes*, *Oscillospira*, *Veillonellaceae* (two ASVs), an unclassified Rikenellaceae RC9 gut group member, *Odoribacter*, two ASVs resolved to *Bacteroides massiliensis*, *Ruminococcaceae*, *Oscillospiraceae* (two ASVs), two *Lachnospiraceae* NK4A136 group ASVs, and three further Clostridia UCG-014 ASVs—broadly overlapping with the G4 vs. G5 caecal signature, consistent with G2 and G5 sharing a postbiotic-supplemented background that differs only in fattening protocol.

##### Post-Mortem Chilling Effect (G1 vs. G3)

Ileum. G3 was discriminated by *Escherichia coli* (two ASVs), an unclassified *Escherichia–Shigella* species, and *Cetobacterium* (LDA 3.4–5.2). G1 was discriminated by *Campylobacter jejuni*, *Streptococcus suis*, *Streptococcus porci*, and *Cetobacterium*.

Caecum. G3 was discriminated by *Lactobacillus johnsonii*, *Streptococcus gallolyticus*, *Slackia*, *Merdibacter*, and an unclassified Clostridia vadinBB60 group member (LDA 2.5–4.6). G1 was discriminated by *Bacteroides* (the strongest effect, −4.51, plus three further ASVs), *Faecalibacterium* (two ASVs), *Christensenellaceae* R-7 group, *Colidextribacter*, *Rikenella*, *Ruminiclostridium*, and an unclassified NK4A214 group member.

### 3.4. Alpha Diversity

**Figure 7:**
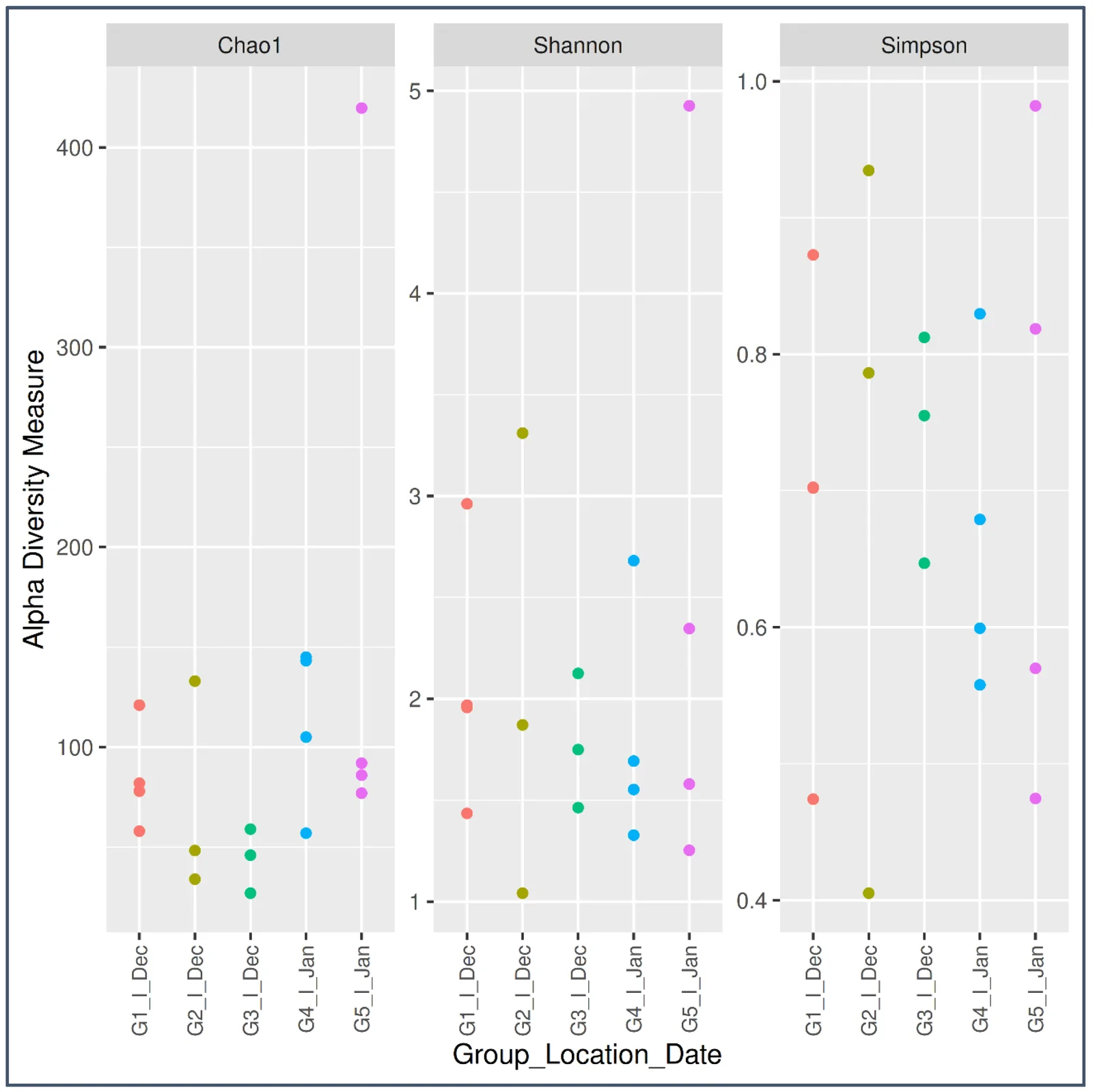
Alpha diversity of the ileal microbiota across the five experimental groups (G1–G5). Community richness was assessed using the Chao1 index, while community diversity was evaluated using the Shannon and Simpson indices. Each point represents one biological replicate.

*Ileum.* Alpha diversity indices in the ileum were consistently lower than in the caecum across all five groups (Figure 7), consistent with the comparatively simple, transit-dominated microbial community typical of the small intestine. Chao1 richness ranged from approximately 35 to 140 across G1–G4, with a single high-richness outlier in G5 (~420) driving a wider spread in this group; Shannon and Simpson indices followed a similar pattern, with G2_I showing the lowest and most variable diversity of the five groups. No clear, consistent separation between December (G1–G3) and January (G4, G5) cohorts was apparent in the ileum across any of the three indices.

**Figure 8:**
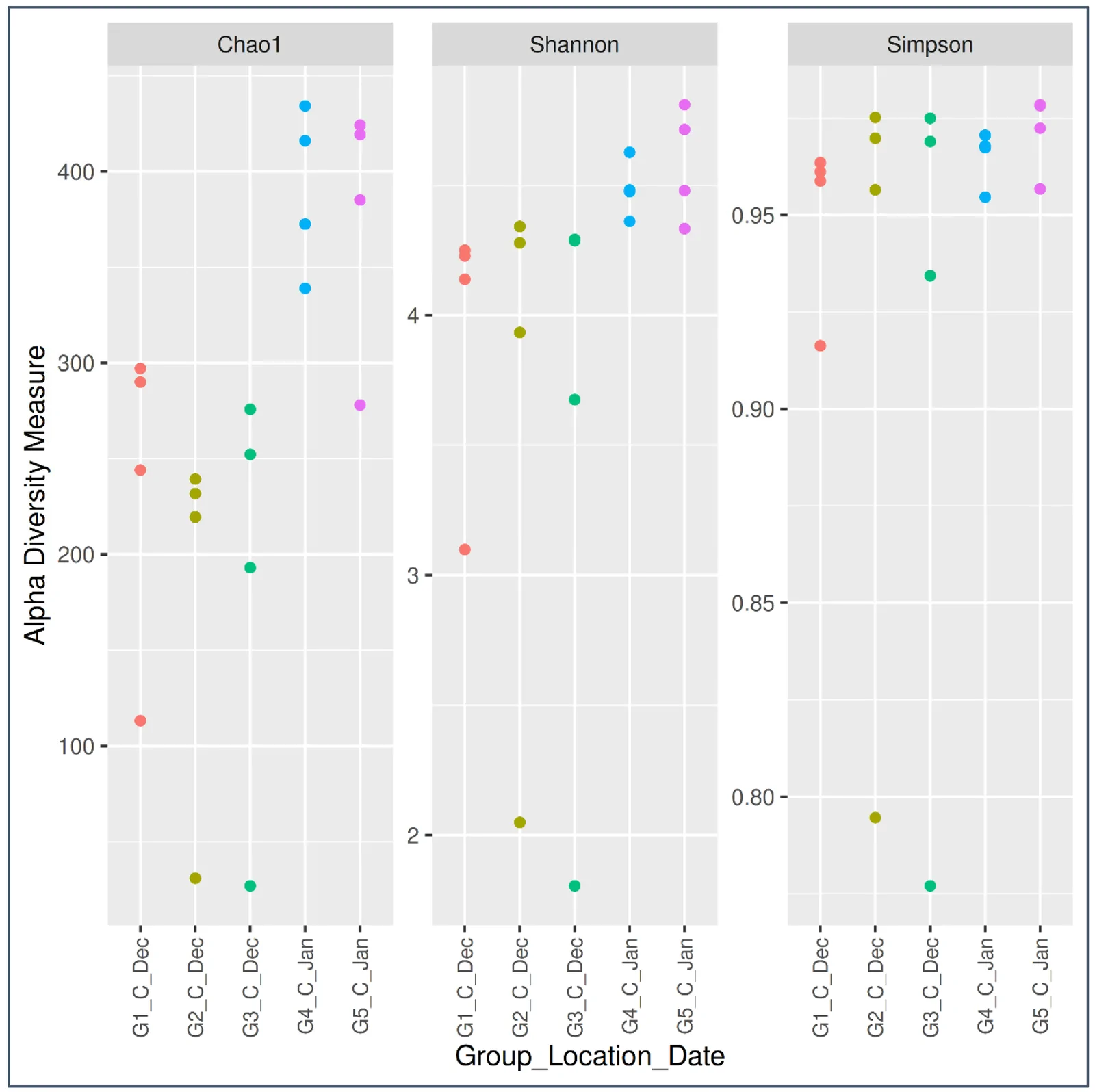
Alpha diversity of the caecal microbiota across the five experimental groups (G1–G5). Community richness was assessed using the Chao1 index, while community diversity was evaluated using the Shannon and Simpson indices. Each point represents one biological replicate.

*Caecum.* Alpha diversity was markedly higher and more stable in the caecum than in the ileum across all indices (Figure 8), as expected given the greater fermentative capacity and residence time of this segment. A visual trend toward higher richness and diversity in the January cohort (G4, G5; Chao1 roughly 280–425) relative to the December cohort (G1–G3; Chao1 roughly 115–300) was apparent across all three indices, mirroring the fattening-protocol-driven separation observed in beta diversity (Section 3.5) and LEfSe (Section 3.3.5).

*Statistical comparison***.** Pairwise differences in alpha diversity between the ten pre-specified group/segment comparisons were assessed using the Conover–Iman test with Benjamini–Hochberg correction (primary analysis), with Wilcoxon rank-sum and Nemenyi tests reported as sensitivity comparisons (Supplementary Table S2). In the ileum, no comparison reached statistical significance with any method (Conover–Iman-adjusted *p* ≥ 0.093). In the caecum, most comparisons likewise remained non-significant; however, G2 vs. G5 showed a statistically significant difference in both Shannon diversity (Conover–Iman-adjusted *p* = 0.037) and Chao1 richness (adjusted *p* = 0.012), though not in Simpson evenness (adjusted *p* = 0.096). This result was not detected by the more conservative Wilcoxon or Nemenyi tests (both adjusted *p* > 0.6) consistent with their known conservatism at this sample size; given that the Conover–Iman test is specifically recommended for small, unequal group sizes such as those used here, we consider it the more appropriate primary test. This finding indicates that postbiotic-supplemented birds differed in caecal alpha diversity between the two fattening protocols (G2: force-fed; G5: restricted-fed). Notably, the same G2 vs. G5 caecal comparison also had the numerically strongest (though not statistically significant after correction) PERMANOVA result among the ten beta-diversity comparisons (R^2^ = 0.372, raw *p* = 0.029, adjusted *p* ≈ 0.074–0.095; Section 3.5.2), suggesting a consistent, if modest, biological signal across both types of analysis. All other pairwise comparisons across both segments remained non-significant after correction (lowest adjusted *p* = 0.067, G1 vs. G4 caecum, Chao1). Thus, while fattening protocol was associated with pronounced shifts in community structure (beta diversity) and in the relative abundance of individual taxa (LEfSe), alpha diversity showed a statistically robust difference only for the G2 vs. G5 caecal comparison once corrected for multiple testing.

### 3.5. Beta Diversity and Community-Level Statistical Comparisons

#### 3.5.1. Overall Community Structure (PCoA)

**Figure 9:**
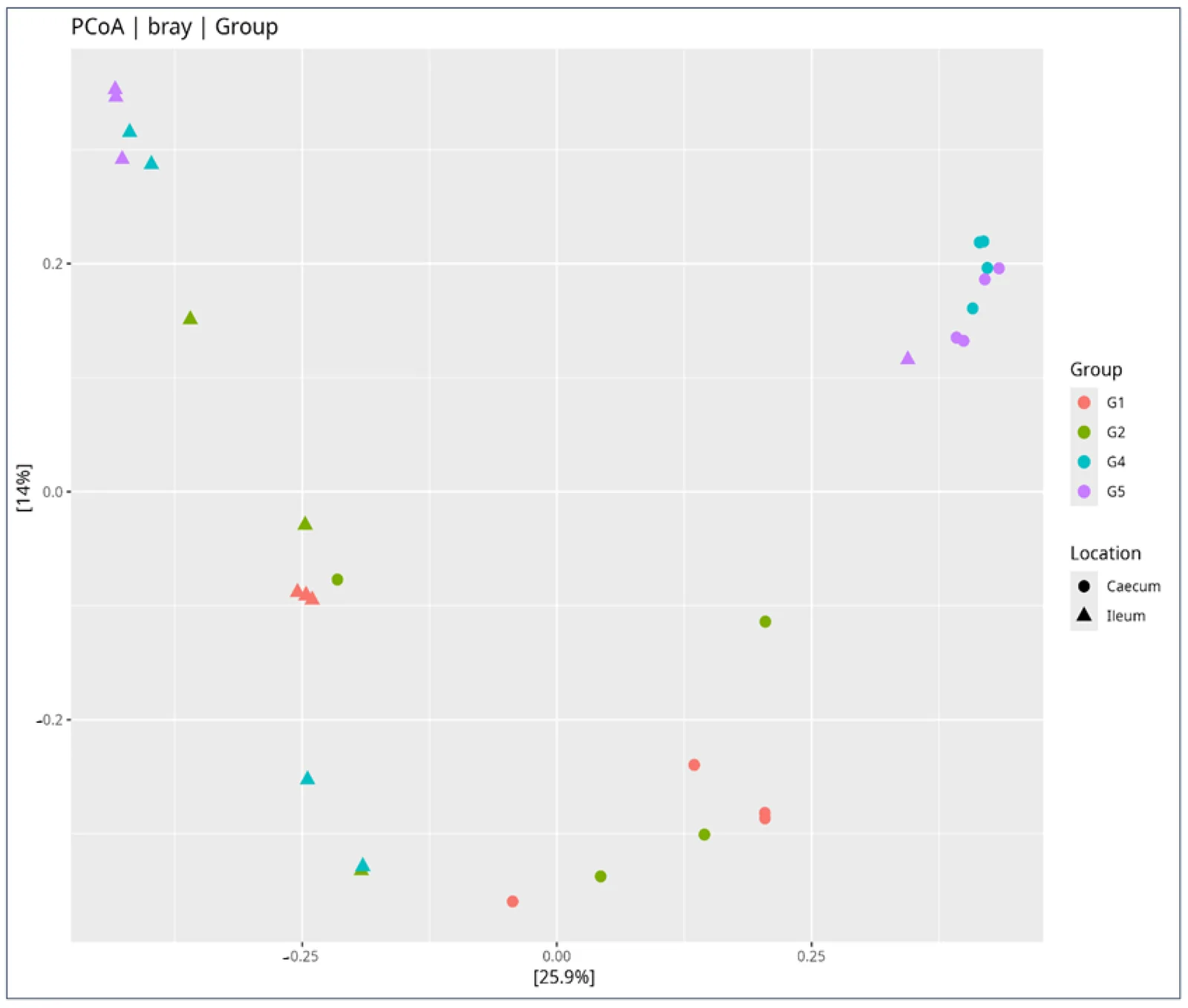
Beta-diversity plot (method: PCoA distance calc: bray). Ileal and caecal microbiome samples from force-fed control (G1) and force-fed postbiotic-supplemented (G2), restricted-fed control (G4) and restricted-fed postbiotic-supplemented (G5) geese.

Bray–Curtis dissimilarity-based principal coordinates analysis (PCoA) of all 38 samples resolved 25.9% and 14.0% of total variance along the first two axes, respectively (Figure 9). Samples separated primarily according to intestinal segment, with ileal and caecal samples occupying largely distinct regions of ordination space, confirming intestinal location as the dominant structuring factor of microbial community composition. Within each segment, a secondary cohort-related clustering was also apparent, with January cohort samples (G4, G5) forming a more compact cluster than December cohort samples (G1, G2), particularly in the caecum. Segment-specific ordinations of warm-dissected samples are provided in Supplementary Figures S2 and S3. In the ileum-only ordination (Supplementary Figure S2; axis 1: 29.8%, axis 2: 24.7%), December cohort samples (G1, G2) tended to separate from January cohort samples (G4, G5) along axis 1, though with considerable scatter reflecting the high inter-individual variability in the ileal microbiota. In the caecum-only ordination (Supplementary Figure S3; axis 1: 37.9%, axis 2: 15.3%), a markedly clearer separation was observed between December and January cohorts, consistent with cohort- and protocol-related differences in caecal community structure, as reflected in the PERMANOVA results for G1 vs. G4 (R^2^ = 0.513, *p* = 0.029) and G2 vs. G5 (R^2^ = 0.372, *p* = 0.029)—though neither remained significant after Benjamini–Hochberg correction across all ten comparisons.

#### 3.5.2. PERMANOVA

Pairwise PERMANOVA (adonis2, Bray–Curtis dissimilarity, 9999 permutations) was performed separately for ileal and caecal samples across five planned comparisons (Supplementary Table S2). Fattening protocol emerged as the strongest and most consistent driver of beta diversity: G1 vs. G4 comparison was significant in both ileum (R^2^ = 0.363, *p* = 0.0286) and caecum (R^2^ = 0.513, *p* = 0.0286), the latter representing the largest effect size observed across all comparisons. A similar pattern was evident for G2 vs. G5, which was significant in caecum (R^2^ = 0.372, *p* = 0.0286) though only trend-level in ileum (*p* = 0.0857); as this comparison contrasts postbiotic-supplemented geese under the two different fattening protocols, it likely reflects the same protocol-driven effect rather than an independent postbiotic signal. In contrast, postbiotic supplementation within a given fattening protocol did not produce a statistically significant shift in overall community structure: neither G1 vs. G2 (force-feeding; ileum *p* = 0.0857, caecum *p* = 0.5143) nor G4 vs. G5 (restricted feeding; ileum *p* = 0.2286, caecum *p* = 0.0571) reached significance, although the latter showed a trend in caecum. This indicates that, at the level of overall beta diversity, postbiotic supplementation exerted a comparatively subtle effect relative to fattening protocol—consistent with the taxon-specific, rather than community-wide, differences identified by LEfSe (Section 3.3.5). Post-mortem chilling (G1 vs. G3) showed a trend-level effect in the ileum (R^2^ = 0.470, *p* = 0.0571) but not in the caecum (*p* = 0.2571), suggesting that apparent microbial community composition in the ileum may be more susceptible to post-mortem/storage-related change than in the caecum over the 24 h chilling period tested (Supplementary Table S3). Homogeneity of multivariate dispersion (PERMDISP) was confirmed for all ten comparisons prior to interpreting these PERMANOVA results (all Pr(>F) ≥ 0.108; full values in Supplementary Table S2), indicating that the observed and trend-level differences reflect shifts in group centroids rather than in within-group dispersion. As these ten comparisons were tested together, we additionally applied Benjamini–Hochberg correction across all ten PERMANOVA *p*-values; none remained statistically significant after correction (adjusted *p* ≥ 0.095). Given the small group sizes (n = 3–4) and the pre-specified, hypothesis-driven nature of these five planned comparisons per segment, we report the uncorrected *p*-values as the primary results in the main text, consistent with treating this analysis as hypothesis-generating rather than confirmatory, while the FDR-adjusted values are provided in Supplementary Table S2 for transparency.

### 3.6. Shared and Unique ASVs Across Groups—Core Microbiome Analysis

**Figure 10:**
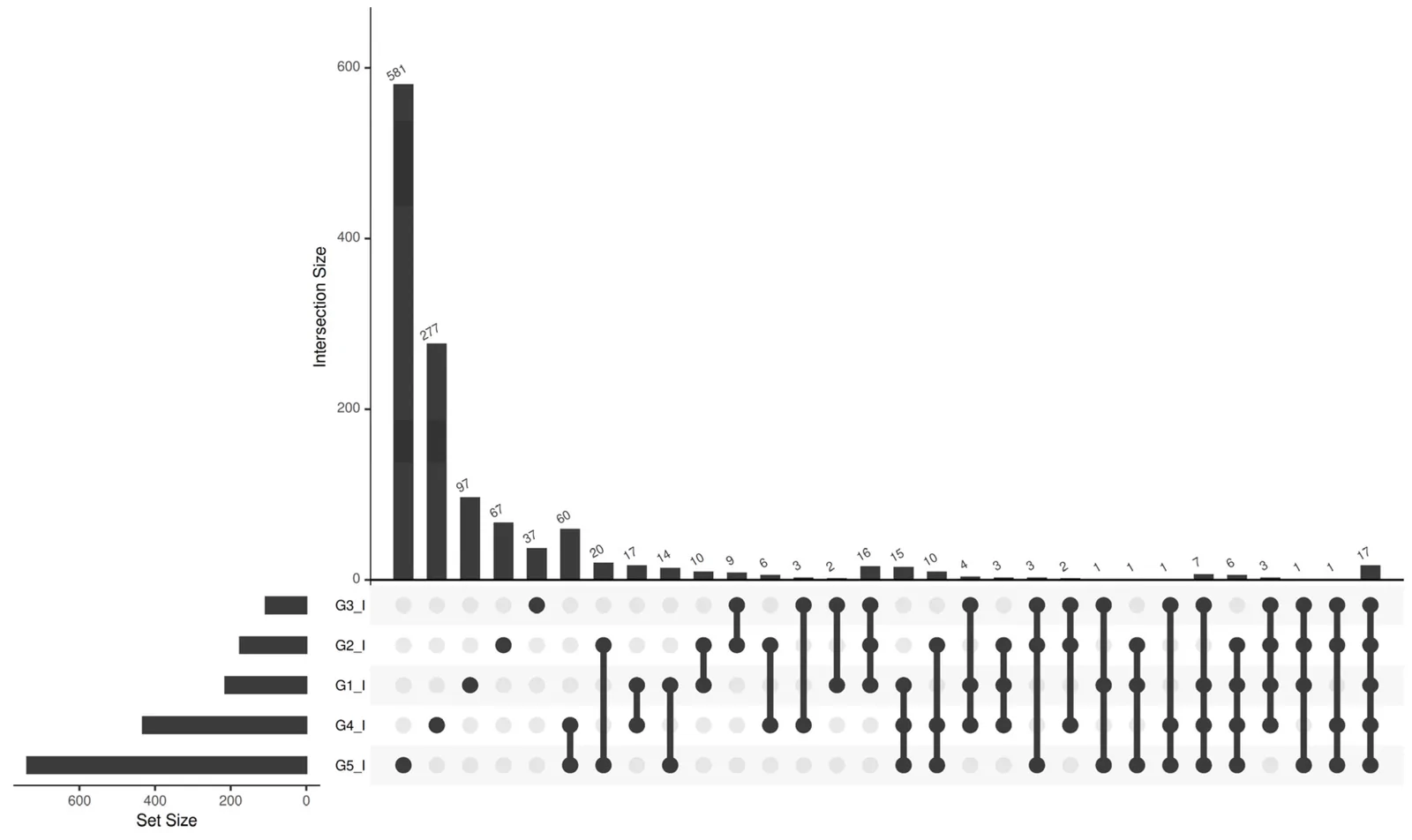
UpSet plot showing shared and unique amplicon sequence variants (ASVs) among ileal samples of groups G1–G5. Set size bars (left) indicate the total number of ASVs detected in each group; intersection bars (top) indicate the number of ASVs shared exclusively by the indicated combination of groups.

**Figure 11:**
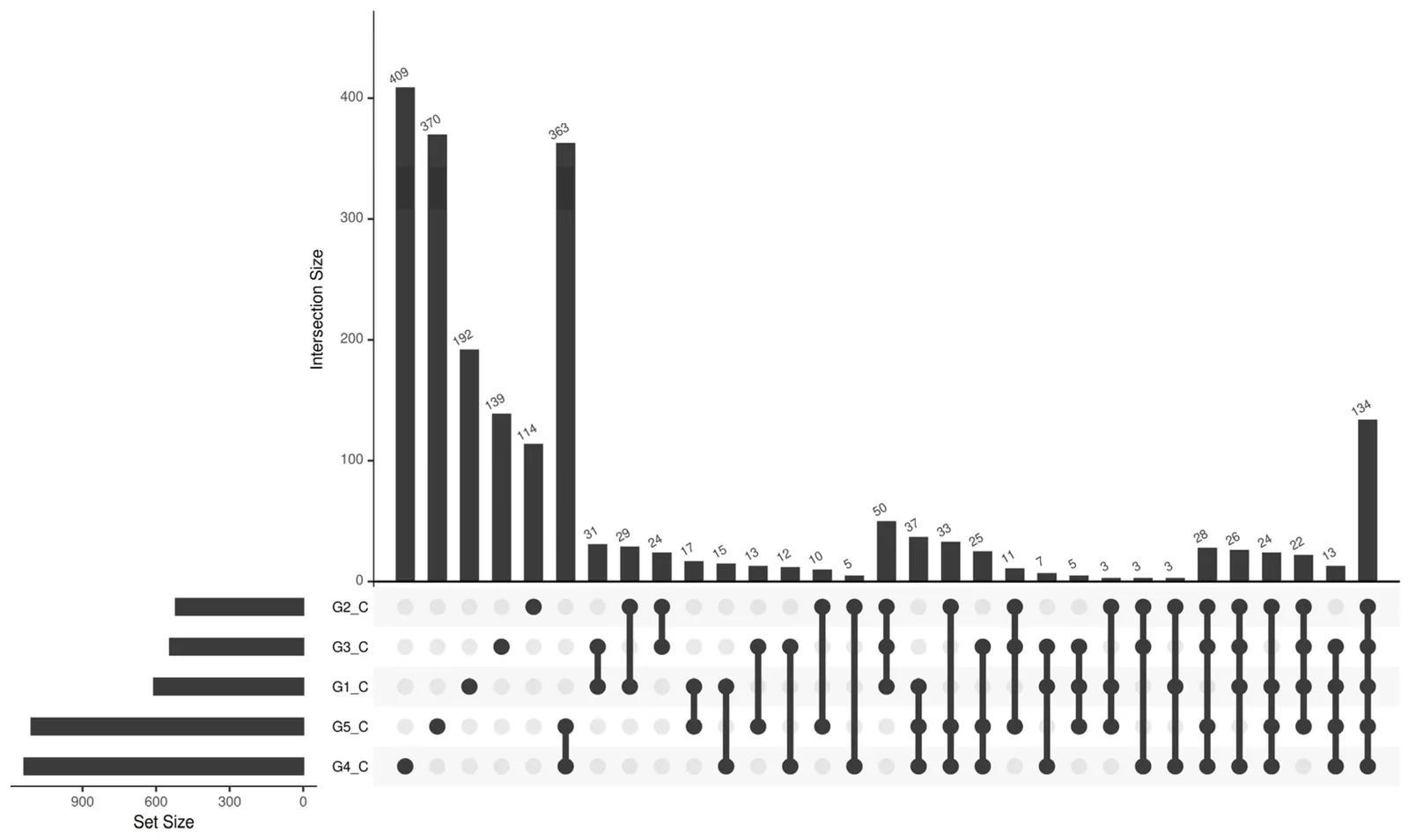
UpSet plot showing shared and unique amplicon sequence variants (ASVs) among caecal samples of groups G1–G5. Set size bars (left) indicate the total number of ASVs detected in each group; intersection bars (top) indicate the number of ASVs shared exclusively by the indicated combination of groups.

The distribution of the amplicon sequence variants (ASVs) among the five experimental groups was visualised separately for the ileum and caecum using segment-specific UpSet plots to evaluate community membership. In the ileum (Figure 10), the community structure was heavily dominated by group-unique taxa, particularly within the January cohort: 581 ASVs were unique to G5_I and 277 to G4_I. The December groups harboured noticeably fewer unique variants, with 97 (G1_I), 67 (G2_I), and 37 (G3_I) ASVs restricted to single groups. Only 17 ASVs were shared across all five ileal groups, indicating a substantially smaller and more transient core community in the ileum than in the caecum. This aligns with the ileum’s physiological role as a transit-dominated intestinal segment characterized by a less stable resident microbiota. In the caecum (Figure 11), the structural partitioning of group-unique ASVs strongly reflected a protocol- and cohort-related contrast, with the largest single-group-specific sets belonging exclusively to the January cohort: 409 ASVs were unique to G4_C and 370 to G5_C. In comparison, the December groups contributed substantially fewer unique variants, with 192, 139, and 114 ASVs being unique to G1_C, G3_C, and G2_C, respectively.

This distribution closely mirrors the higher Chao1 richness values observed in the January cohort. A total of 134 ASVs were shared across all five caecal groups, representing a robust, putative core caecal microbiota that remains stable regardless of the fattening protocol or postbiotic supplementation. Overall, the substantially larger core set in the caecum (134 vs. 17 ASVs) relative to its total richness underscores the greater stability and resilience of the caecal microbial community compared to the ileum. The heavy concentration of group-unique ASVs in the January cohort across both intestinal segments further reinforces fattening protocol—rather than postbiotic supplementation—as the primary driver of community membership.

## 4. Discussion

The present study provides the first paired ileum–caecum characterization of the gut microbiota in geese subjected to contrasting fattening protocols with or without Burain^®^ postbiotic supplementation, and additionally evaluates the methodological impact of post-mortem chilling on apparent microbiome composition. Three main findings emerged: (i) cohort- and protocol-related differences were associated with the largest shifts in gut microbial community structure, particularly in the caecum; (ii) postbiotic supplementation produced taxon-specific, segment-dependent shifts that were detectable under restricted feeding but not under force-feeding conditions; and (iii) post-mortem chilling substantially altered the ileal microbiota, with more limited effects in the caecum. The most consistent pattern in this study was a cohort- and protocol-related difference in caecal and ileal microbiota between the December and January groups. PERMANOVA revealed significant differences between force-fed (G1) and restricted-fed (G4) control animals in both the ileum (R^2^ = 0.363, *p* = 0.029) and the caecum (R^2^ = 0.513, *p* = 0.029), the latter representing the largest effect size observed across all comparisons. The December and January cohorts differed not only in fattening protocol but also in age at slaughter (~10 vs. ~14 weeks) and duration of postbiotic exposure; these confounding factors cannot be fully disentangled in the present design, and the observed differences should therefore be attributed to the combined effect of fattening protocol, age, and supplementation history, rather than to any single variable in isolation. This limitation is common to comparative production studies conducted under real farm conditions of meat-producing swan goose [7]. These results are consistent with previous reports demonstrating that overfeeding substantially disrupts goose intestinal microbiota, reducing caecal microbial diversity and shifting community composition toward dysbiotic profiles enriched in *Escherichia–Shigella* and depleted in beneficial Clostridia [5,6]. In the present study, force-fed animals (G1) showed ileal dominance of *Romboutsia*, *Clostridium*, and *Helicobacter*, whereas restricted-fed animals (G4) were characterized by high but variable *Escherichia–Shigella* and *Ligilactobacillus* abundances. In the caecum, G4 showed elevated *Christensenellaceae*, *Oscillospira*, and multiple Clostridia UCG-014 members relative to G1, while G1 retained higher *Prevotellaceae*, *Alistipes*, and *Bilophila*. The enrichment of *Christensenellaceae* in the restricted-fed group is particularly noteworthy, as members of this family have been consistently associated with host metabolic health, lean body composition, and microbiome stability across mammalian and avian species [31]. The caecal *Oscillospiraceae* complex (including *Oscillospira*, NK4A214 group, and UCG-005) was also elevated in G4, consistent with a more mature fermentative community associated with fiber degradation and short-chain fatty acid (SCFA) production [1] which was detected in *Anser cygnoides.* It should be noted that the December and January cohorts differed not only in fattening protocol but also in age at slaughter (~10 vs. ~14 weeks) and duration of postbiotic exposure.

### 4.1. Postbiotic Effects: Protocol-Dependent and Segment-Specific

Postbiotic supplementation with Burain^®^ did not produce a significant shift in overall community structure as assessed by PERMANOVA in either intestinal segment under force-feeding conditions (G1 vs. G2: ileum *p* = 0.086, caecum *p* = 0.514). Under restricted feeding, a trend-level effect was observed in the caecum (G4 vs. G5: *p* = 0.057, R^2^ = 0.223) but not in the ileum (*p* = 0.229). These results suggest that the effect of postbiotic supplementation on overall beta diversity is modest and context-dependent, consistent with findings in broiler chickens where postbiotic-associated microbiome shifts were subtle at the community level but detectable at the taxon level [32]. As the overall PERMANOVA comparisons for postbiotic supplementation did not reach statistical significance in either segment, and the underlying group sizes were small (n = 3–4), the following taxon-level LEfSe patterns should be interpreted as exploratory rather than confirmatory evidence of a postbiotic effect. At the taxon level, LEfSe analysis revealed protocol-specific postbiotic signatures. Under force-feeding (G1 vs. G2), the postbiotic-supplemented group showed enrichment of *Ligilactobacillus* and *Megamonas* in the ileum, and *Streptococcus gallolyticus* and *Butyricimonas* in the caecum. Under restricted feeding (G4 vs. G5), G5 showed ileal enrichment of *Romboutsia* and *Christensenellaceae*, and caecal enrichment of *Bacteroides dorei*, *Lachnospiraceae* NK4A136 group, *Alistipes*, and multiple Clostridia UCG-014 members—a profile broadly consistent with a more diverse, fermentation-active community. The near-absence of *Escherichia–Shigella* in G5 ileum relative to G4 (0.05% vs. 34.86%) is particularly relevant from a gut health perspective, as this genus encompasses both commensal and pathogenic strains whose enrichment has been linked to intestinal dysbiosis and disrupted bile acid metabolism in overfed Landes geese [5]. Interestingly, the response of *Escherichia–Shigella* to postbiotic supplementation appeared to be highly dependent on the feeding regime. While its abundance drastically decreased under restricted feeding, it showed a notable, albeit highly variable, increase in the force-fed group (G2: 39.62% vs. G1: 0.80%). This suggests that the profound environmental shift caused by extreme carbohydrate loading may alter the direction of postbiotic–microbiome interactions.

The observation that postbiotic effects were more detectable under restricted feeding, where supplementation was continuous through slaughter, than under force-feeding, where it was discontinued for 14 days prior to sampling, suggests a possible priming effect, which we propose here as a hypothesis for future longitudinal studies rather than as an established mechanism. Postbiotic supplementation during the rearing phase may have promoted microbiome resilience, enabling the intestinal community to better maintain a beneficial compositional profile under the metabolic stress of intensive feeding. This interpretation is consistent with the concept of microbiome resilience, whereby prior colonization history and dietary conditioning influence the community’s response to perturbation in broiler chicken [33]. However, as intestinal samples were collected after rather than before the overfeeding period, direct mechanistic evidence for this carry-over effect remains to be established in future longitudinal studies. The concurrent observation that postbiotic-supplemented animals in the January cohort had significantly darker and less yellow livers (lower L* and b* values) without differences in liver weight or stiffness is consistent with Szabó et al. [14], who reported the same L* and b* pattern in the force-fed December cohort, suggesting a robust and protocol-independent effect of Burain^®^ on liver colour parameters—possibly through modulation of lipid metabolism or hepatic pigment deposition [34].

The warm- and chilled-dissection groups differed in ileal but not caecal microbiota. Post-mortem chilling for 24 h at 4 °C produced a trend-level shift in ileal community composition (G1 vs. G3: R^2^ = 0.470, *p* = 0.057) but not in the caecum (*p* = 0.257). As the warm-dissected (G1) and chilled-dissected (G3) birds were different individuals, individual-animal variation cannot be separated from the effect of chilling in this comparison; the result should therefore be interpreted as exploratory. At the genus level, the chilled ileum showed marked enrichment of *Escherichia–Shigella*, *Lactobacillus*, *Ligilactobacillus*, *Campylobacter*, and *Gallibacterium* relative to the warm-dissected control, while *Romboutsia*, *Helicobacter*, and *Cetobacterium* were reduced. These shifts are consistent with the known cold-tolerant growth advantage of facultative anaerobes and psychrotrophic bacteria at refrigeration temperatures [16], and suggest that apparent ileal microbiome composition in geese may be substantially influenced by post-mortem handling conditions. The relative stability of caecal composition under chilling may reflect the higher microbial density and community resilience of this segment, consistent with the buffering capacity attributed to the caecal fermentation environment in swan geese [1]. From a methodological standpoint, these findings underscore the importance of standardizing dissection timing in goose microbiome studies, particularly for ileal samples, and caution against direct comparisons between studies using warm and chilled carcass protocols.

### 4.2. Alpha Diversity

Using the Conover–Iman post hoc test, which is better suited than the Wilcoxon or Nemenyi tests to the small, unequal group sizes used here, caecal richness (Shannon, Chao1) differed significantly between the G2 and G5 postbiotic-supplemented groups reared under different fattening protocols, although Simpson diversity did not differ, suggesting a change in richness/rarity rather than in dominance structure. This same G2 vs. G5 (caecum) contrast also had the numerically strongest PERMANOVA result among the beta-diversity comparisons, although it did not reach significance there after correction (Section 3.5.2; Supplementary Table S2). Taken together, these results suggest a modest but reasonably consistent postbiotic-associated difference in caecal community richness between the two fattening protocols, detectable in overall richness before it is reflected in a statistically significant shift in overall community composition. No other pairwise comparison reached significance for alpha diversity in either segment. Given the small sample size, we regard this as a preliminary finding warranting confirmation in a larger, adequately powered study. Limitations and future directions are as follows.

The primary limitation of this study is the small sample size per group (n = 3–4), no a priori power calculation was performed, and the resulting low statistical power should be borne in mind when interpreting all non-significant and trend-level results reported throughout this study, not only those explicitly framed as PERMANOVA or alpha diversity trends. For the same reason, individual LEfSe taxon-level findings—which are not corrected for multiple comparisons—are best weighted alongside the community-level (PERMANOVA) and descriptive abundance results reported elsewhere, rather than interpreted in isolation. The confounded design—in which fattening protocol, age, and postbiotic exposure duration co-vary between cohorts—prevents unambiguous attribution of observed differences to a single factor. Future studies should incorporate larger group sizes, pre-fattening baseline sampling, and ideally a factorial design separating fattening protocol from supplementation duration. The addition of functional analyses (metagenomics, metabolomics) would further clarify whether the taxon-level shifts identified here translate into differences in SCFA production, bile acid profiles, or immune function relevant to goose health and foie gras quality.

## 5. Conclusions

This study provides the first paired ileum–caecum characterization of the gut microbiota in geese receiving Burain^®^ postbiotic supplementation under two contrasting fattening protocols. Fattening protocol (confounded with cohort, age at slaughter, and duration of postbiotic exposure) was associated with the largest differences in microbial community structure, producing significant beta diversity shifts in both intestinal segments, with the strongest effect observed in the caecum. Postbiotic supplementation exerted taxon-specific, segment-dependent effects that were detectable under continuous restricted feeding conditions but were not apparent following force-feeding, during which supplementation had been discontinued. This pattern suggests that the detectability of postbiotic-associated microbiome modulation depends on both the continuity of supplementation and the magnitude of protocol-imposed community disruption. Postbiotic supplementation was associated with differences in liver colour, while liver weight and stiffness were unchanged; the biological basis of this finding requires further study. From a methodological perspective, the warm- and chilled-dissection groups differed in apparent ileal microbiota (individual-animal variation cannot be separated from the effect of chilling in this comparison, as the two groups consisted of different birds), while caecal composition remained comparatively stable. This finding highlights the importance of standardizing dissection timing in goose microbiome research, particularly for ileal samples. Taken together, these results advance the understanding of how nutritional interventions and production conditions shape the goose gut microbiome, and provide a reference framework for designing postbiotic strategies that account for fattening protocol and intestinal segment specificity.

## Figures and Tables

**Table 1:** Composition of starter and grower feeds.

	Starter (Days 1–21)	Grower (Days 22–56)
Moisture content %	14.00	14.00
Crude protein %	20.00	18.00
Crude fat %	3.00	3.70
Crude fibre %	4.30	5.40
Crude ash %	5.62	5.00
Lysine %	1.06	0.97
Methionine %	0.50	0.46
Calcium %	1.00	0.85
Total phosphorus %	0.73	0.58
Sodium %	0.15	0.15
Vitamin A, NE/kg	10,000.00	10,000.00
Vitamin D3, NE/kg	3750.00	3750.00
Hy-D3, NE/kg	0.00	0.00
Vitamin E, NE/kg	50.00	50.00

**Table 2:** Composition of feed during the restricted-feeding period.

Self-Fattening (Week 9–14)	
Moisture content %	14.00
Crude protein %	9.55
Crude fat %	9.45
Crude fibre %	3.35
Crude ash %	3.30
Lysine %	0.28
Methionine %	0.17
Calcium %	0.49
Total phosphorus %	0.36
Sodium %	0.22

**Table 3:** Summary of experimental groups.

Group	Cohort	Treatment	Fattening Protocol	Dissection	n (Ileum)	n (Caecum)
**G1**	December	Control	Force-feeding	Warm	4	4
**G2**	December	2 g/kg Burain^®^	Force-feeding	Warm	3	4
**G3**	December	Control	Force-feeding	Chilled (24 h, 4 °C)	3	4
**G4**	January	Control	Restricted feeding	Warm	4	4
**G5**	January	2 g/kg Burain^®^	Restricted feeding	Warm	4	4

Note: G3 is not an independent treatment subgroup but represents additional birds from the G1 population, sampled by chilled rather than warm dissection.

**Table 4:** Live body weight (g) of control (G4) and postbiotic-supplemented (G5) geese during the restricted feeding period (weeks 9–14).

	Live Body Weight (g)	
Week	G4	G5
9	5053.9 ± 510.5 a	5028.4 ± 484.7 a
10	5350.5 ± 631.2 a	5266.7 ± 629.0 a
11	5587.6 ± 707.1 a	5465.6 ± 663.6 a
12	5721.7 ± 731.6 a	5621.7 ± 660.4 a
13	6117.3 ± 1046.9 a	5826.0 ± 867.8 a
14	5936.1 ± 1086.0 a	5889.5 ± 902.9 a

**Table 5:** Slaughter performance and liver quality parameters of control (G4) and postbiotic-supplemented (G5) geese following the restricted feeding protocol.

Parameters	G4	G5
Liver weight (g)	101.1 ± 79.2 a	81.1 ± 23.7 a
L*	43.5 ± 13.4 a	35.7 ± 3.2 b
a*	12.6 ± 2.4 a	12.5 ± 1.9 a
b*	15.6 ± 6.9 a	11.5 ± 2.5 b
Stiffness (g)	391.21 ± 131.0 a	392.39 ± 122.1 a
Breast weight (g)	286.4 ± 55.6 a	273.3 ± 51.7 a
Thigh weight (g)	412.4 ± 72.5 a	407.6 ± 78.8 a

Values are presented as means ± SD. Sample size: n = 59 (G4) and n = 57 (G5) for liver weight; n = 20 per group for all other parameters. a, b: Different superscript letters within the same row indicate significant differences between groups (*p* ≤ 0.05, one-way ANOVA with Tukey HSD post-hoc test).

## Data Availability

The raw sequencing data generated during the current study have been deposited in the NCBI Sequence Read Archive (SRA) database under BioProject accession number PRJNA1506723. Other datasets used and/or analysed during the study are available from the corresponding author on reasonable request.

## Supplementary Materials

The following supporting information can be downloaded at: https://www.mdpi.com/article/10.3390/ani16193153/s1. Figure S1: Linear discriminant analysis Effect Size (LEfSe/lefser) barplots showing significantly abundant bacterial taxa between experimental groups in the ileum and caecum. Figure S2 and S3: Principal Coordinate Analysis (PCoA) plot based on Bray–Curtis distances representing beta diversity of ileum and caecum samples. Table S1: Relative abundance data of identified bacterial taxa at the phylum and genus levels across experimental groups and intestinal segments. Table S2: Statistical evaluation of microbial diversity metrics across experimental groups. Worksheet 1 (“alpha div statistics I”) presents statistical comparisons for alpha diversity indices (Shannon, Chao1, and Simpson), where bold red text indicates statistically significant differences (*p* < 0.05) based on unadjusted Kruskal–Wallis and post hoc Dunn tests. Worksheet 2 (“alpha div statistics II”) presents results of Conover–Iman test (primary, BH-corrected) with Wilcoxon rank-sum and Nemenyi tests reported as sensitivity comparisons, on rarefied and raw abundance data. Worksheet 3 (“beta div statistics”) contains pairwise PERMANOVA (R^2^, F, uncorrected and BH-corrected P) and PERMDISP results for all ten planned comparisons. Bold text indicates statistically significant results (adjusted *p* < 0.05). Table S3: Summary table of LEfSe (lefser) differential abundance analysis results across pairwise group comparisons.
