# Supplementary material for: A Paired Ileum–Caecum Study of the Goose Gut Microbiome in Response to Fattening Protocol and Postbiotic Supplementation
Source: Animals (Basel). 2026 Oct 8;16(19):3153. doi: 10.3390/ani16193153 (PMC13663337; doi:10.3390/ani16193153)
Supplement: Supplementary file 1 [file animals-16-03153-s001.zip › Supplementary Figure S1_lefser_Animals.pdf]

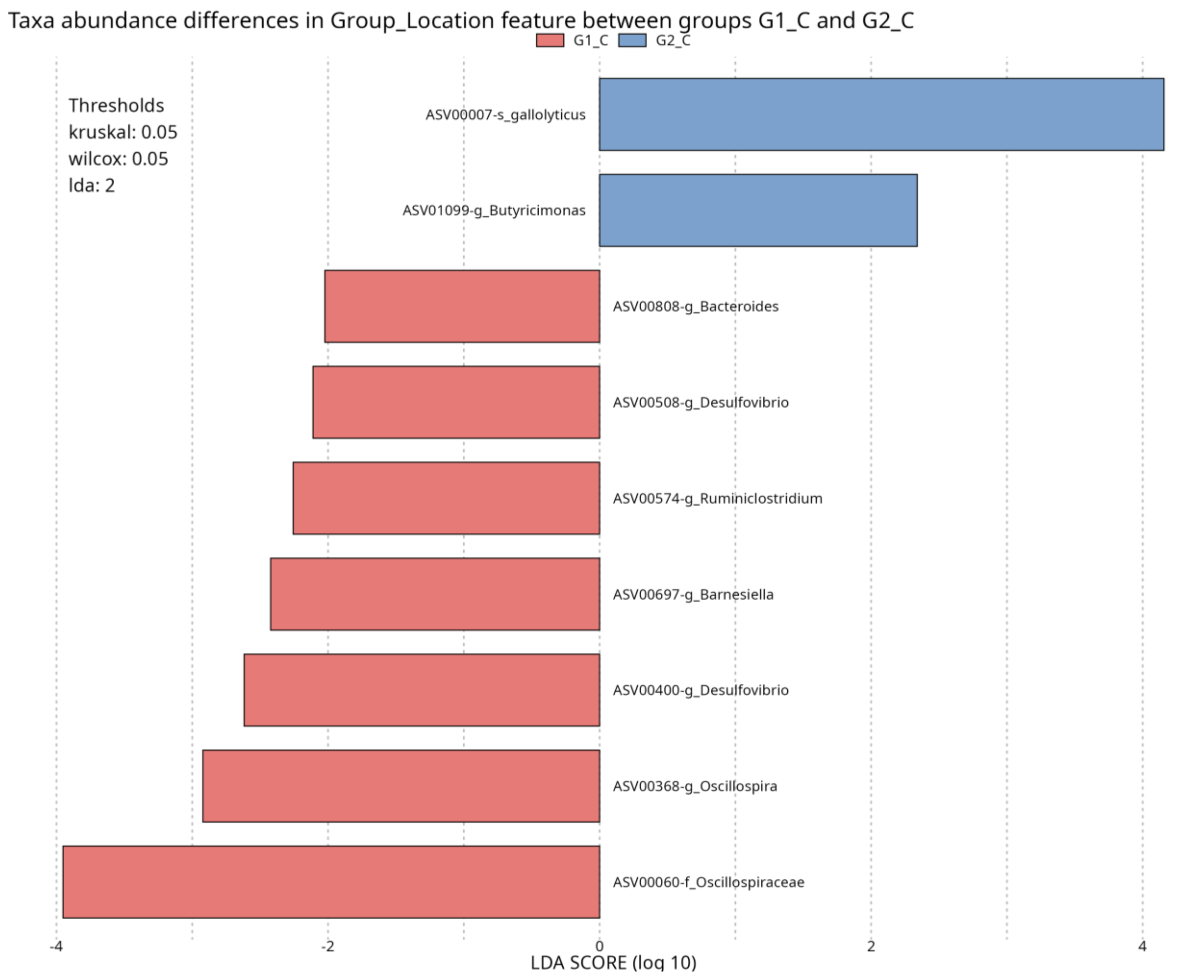
Taxa abundance differences in Group_Location feature between groups G1_C and G2_C
G1_C
G2_C
Thresholds
kruskal: 0.05
wilcox: 0.05
lda: 2
ASV00007-s_gallolyticus
ASV01099-g_Butyricimonas
ASV00808-g_Bacteroides
ASV00508-g_Desulfovibrio
ASV00574-g_Ruminiclostridium
ASV00697-g_Barnesiella
ASV00400-g_Desulfovibrio
ASV00368-g_Oscillospira
ASV00060-f_Oscillospiraceae
-4
-2
0
2
4
LDA SCORE (log 10)

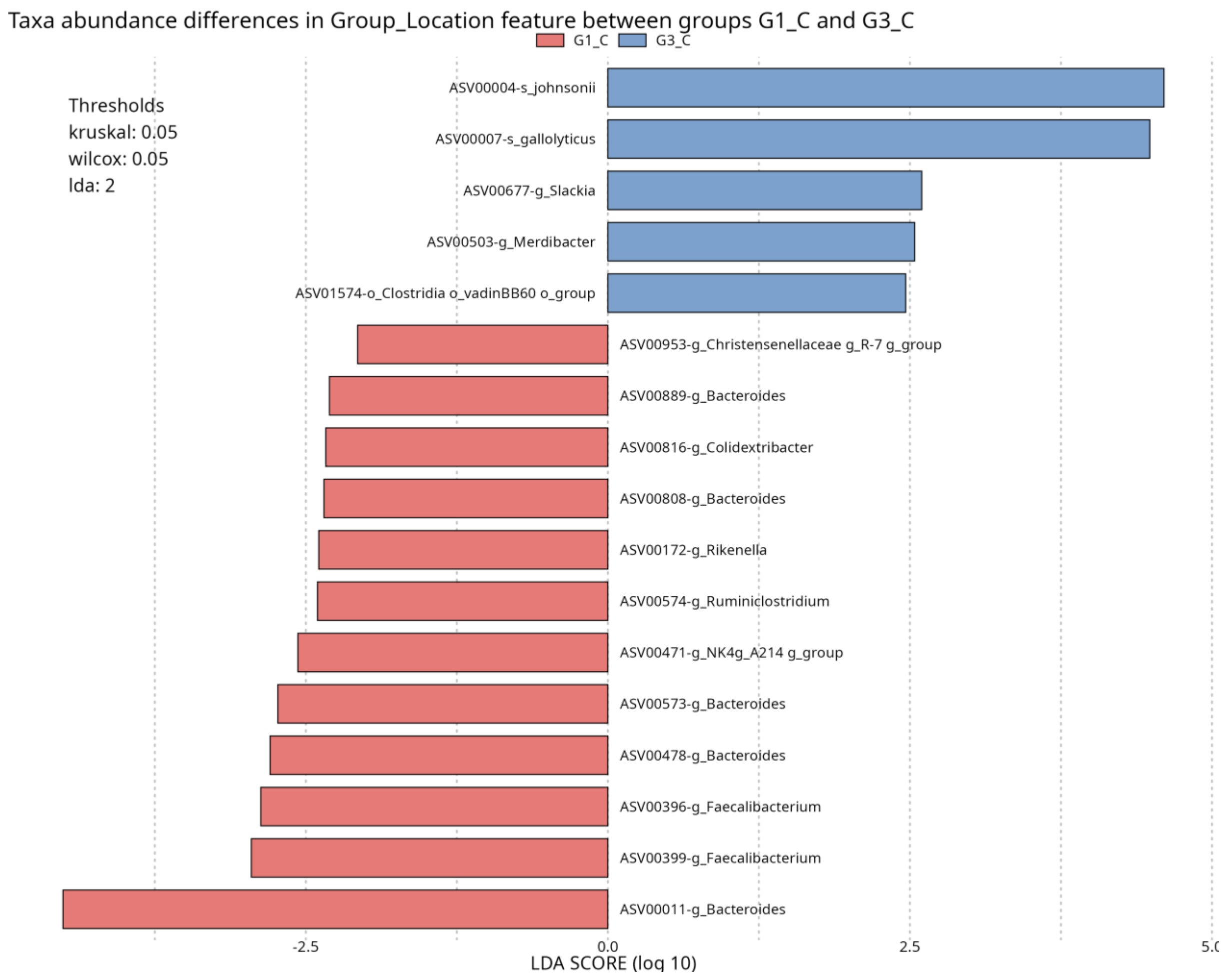
Taxa abundance differences in Group_Location feature between groups G1_C and G3_C
G1_C
G3_C
Thresholds
kruskal: 0.05
wilcox: 0.05
lda: 2
ASV00004-s_johnsonii
ASV00007-s_gallolyticus
ASV00677-g_Slackia
ASV00503-g_Merdibacter
ASV01574-o_Clostridia o_vadinBB60 o_group
ASV00953-g_Christensenellaceae g_R-7 g_group
ASV00889-g_Bacteroides
ASV00816-g_Colidextribacter
ASV00808-g_Bacteroides
ASV00172-g_Rikenella
ASV00574-g_Ruminiclostridium
ASV00471-g_NK4g_A214 g_group
ASV00573-g_Bacteroides
ASV00478-g_Bacteroides
ASV00396-g_Faecalibacterium
ASV00399-g_Faecalibacterium
ASV00011-g_Bacteroides
-2.5
0.0
2.5
LDA SCORE (log 10)

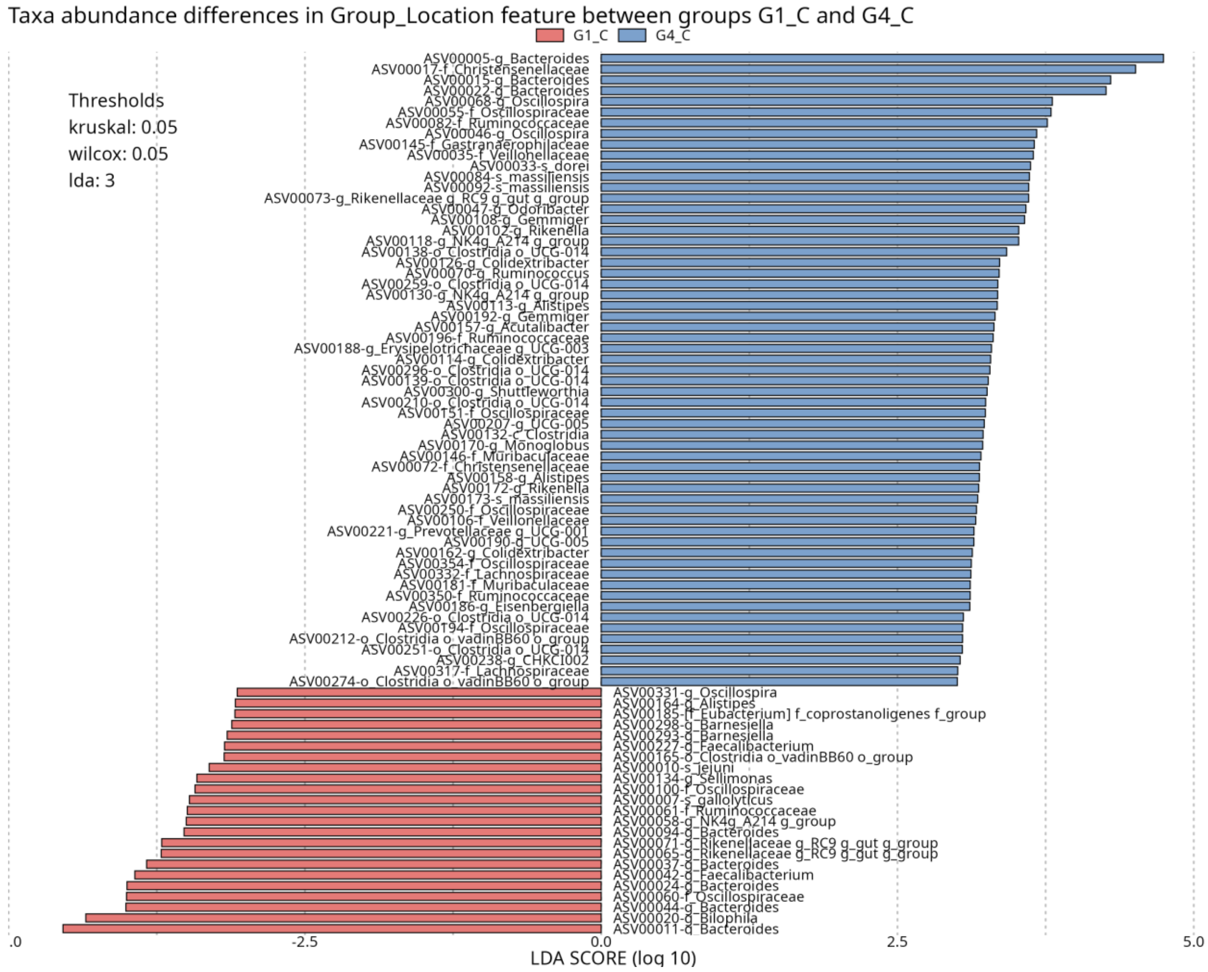
Taxa abundance differences in Group_Location feature between groups G1_C and G4_C
G1_C
G4_C
Thresholds
kruskal: 0.05
wilcox: 0.05
lda: 3
ASV00005-g_Bacteroides
ASV00017-f_Christensenellaceae
ASV00015-g_Bacteroides
ASV00022-g_Bacteroides
ASV00068-g_Oscillospira
ASV00055-f_Oscillospiraceae
ASV00082-f_Ruminococcaceae
ASV00046-g_Oscillospira
ASV00145-f_Gastranaerophilaceae
ASV00035-f_Veillonellaceae
ASV00033-s_dorei
ASV00084-s_massiliensis
ASV00092-s_massiliensis
ASV00073-g_Rikenellaceae g_RC9 g_gut g_group
ASV00047-g_Odoribacter
ASV00108-g_Gemmiger
ASV00102-g_Rikenella
ASV00118-g_NK4g_A214 g_group
ASV00138-o_Clostridia o_UCG-014
ASV00126-g_Colidextribacter
ASV00070-g_Ruminococcus
ASV00259-o_Clostridia o_UCG-014
ASV00130-g_NK4g_A214 g_group
ASV00113-g_Alistipes
ASV00192-g_Gemmiger
ASV00157-g_Acutalibacter
ASV00196-f_Ruminococcaceae
ASV00188-g_Erysipelotrichaceae g_UCG-003
ASV00114-g_Colidextribacter
ASV00296-o_Clostridia o_UCG-014
ASV00139-o_Clostridia o_UCG-014
ASV00300-g_Shuttleworthia
ASV00210-o_Clostridia o_UCG-014
ASV00151-f_Oscillospiraceae
ASV00207-g_UCG-005
ASV00132-c_Clostridia
ASV00170-g_Monoglobus
ASV00146-f_Muribaculaceae
ASV00072-f_Christensenellaceae
ASV00158-g_Alistipes
ASV00172-g_Rikenella
ASV00173-s_massiliensis
ASV00250-f_Oscillospiraceae
ASV00106-f_Veillonellaceae
ASV00221-g_Prevotellaceae g_UCG-001
ASV00190-g_UCG-005
ASV00162-g_Colidextribacter
ASV00354-f_Oscillospiraceae
ASV00332-f_Lachnospiraceae
ASV00181-f_Muribaculaceae
ASV00350-f_Ruminococcaceae
ASV00186-g_Eisenbergiella
ASV00226-o_Clostridia o_UCG-014
ASV00194-f_Oscillospiraceae
ASV00212-o_Clostridia o_vadinBB60 o_group
ASV00251-o_Clostridia o_UCG-014
ASV00238-g_CHKCI002
ASV00317-f_Lachnospiraceae
ASV00274-o_Clostridia o_vadinBB60 o_group
ASV00331-g_Oscillospira
ASV00164-g_Alistipes
ASV00185-[f_Eubacterium] f_coprostanoligenes f_group
ASV00298-g_Barnesiella
ASV00293-g_Barnesiella
ASV00227-g_Faecalibacterium
ASV00165-o_Clostridia o_vadinBB60 o_group
ASV00010-s_jejuni
ASV00134-g_Sellimonas
ASV00100-f_Oscillospiraceae
ASV00007-s_gallolyticus
ASV00061-f_Ruminococcaceae
ASV00058-g_NK4g_A214 g_group
ASV00094-g_Bacteroides
ASV00071-g_Rikenellaceae g_RC9 g_gut g_group
ASV00065-g_Rikenellaceae g_RC9 g_gut g_group
ASV00037-g_Bacteroides
ASV00042-g_Faecalibacterium
ASV00024-g_Bacteroides
ASV00060-f_Oscillospiraceae
ASV00044-g_Bacteroides
ASV00020-g_Bilophila
ASV00011-g_Bacteroides
.0
-2.5
0.0
2.5
5.0
LDA SCORE (log 10)

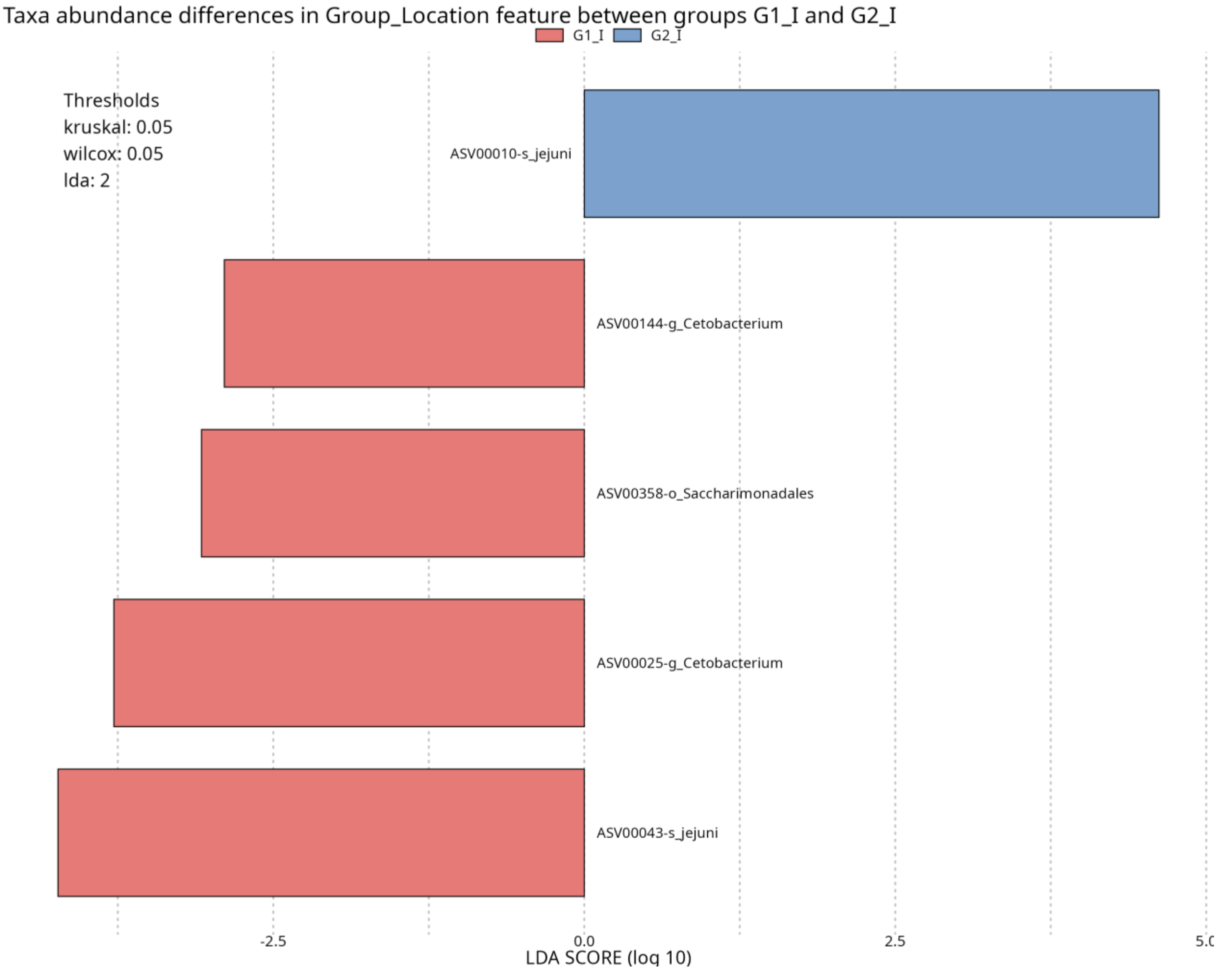
Taxa abundance differences in Group_Location feature between groups G1_I and G2_I
G1_I
G2_I
Thresholds
kruskal: 0.05
wilcox: 0.05
lda: 2
ASV00010-s_jejuni
ASV00144-g_Cetobacterium
ASV00358-o_Saccharimonadales
ASV00025-g_Cetobacterium
ASV00043-s_jejuni
-2.5
0.0
2.5
LDA SCORE (log 10)

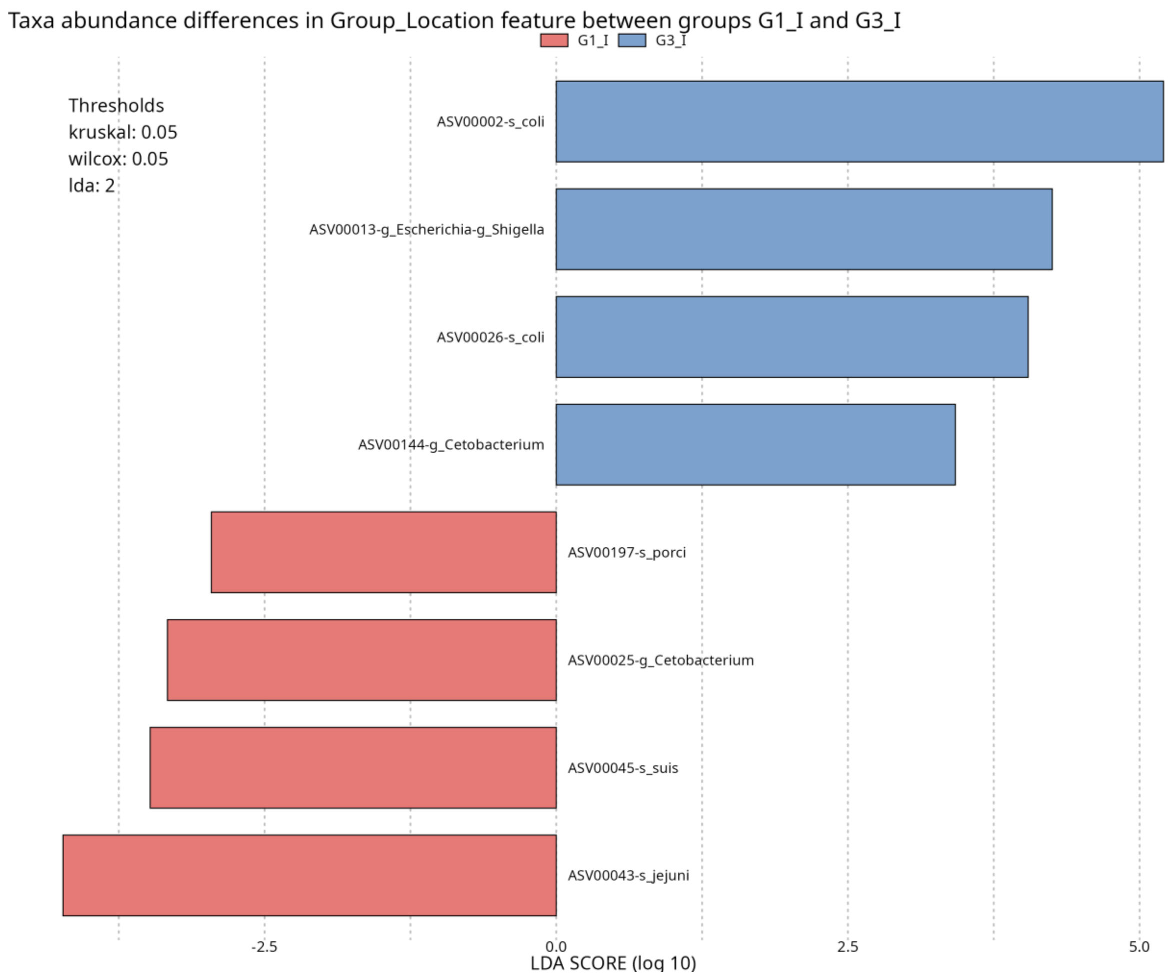
Taxa abundance differences in Group_Location feature between groups G1_I and G3_I
G1_I
G3_I
Thresholds
kruskal: 0.05
wilcox: 0.05
lda: 2
ASV00002-s_coli
ASV00013-g_Escherichia-g_Shigella
ASV00026-s_coli
ASV00144-g_Cetobacterium
ASV00197-s_porci
ASV00025-g_Cetobacterium
ASV00045-s_suis
ASV00043-s_jejuni
-2.5
0.0
2.5
5.0
LDA SCORE (log 10)

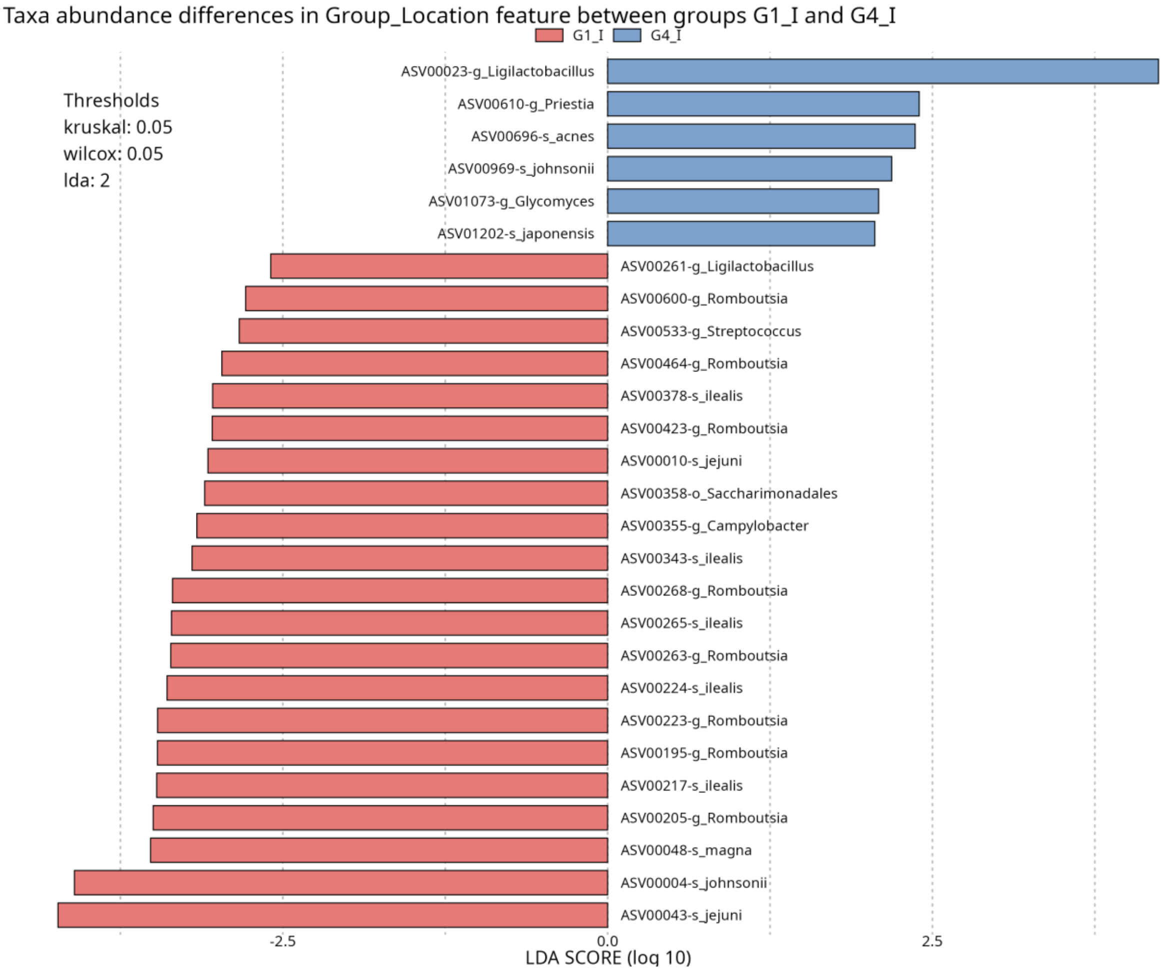
Taxa abundance differences in Group_Location feature between groups G1_I and G4_I
G1_I
G4_I
Thresholds
kruskal: 0.05
wilcox: 0.05
lda: 2
ASV00023-g_Ligilactobacillus
ASV00610-g_Priestia
ASV00696-s_acnes
ASV00969-s_johnsonii
ASV01073-g_Glycomyces
ASV01202-s_japonensis
ASV00261-g_Ligilactobacillus
ASV00600-g_Romboutsia
ASV00533-g_Streptococcus
ASV00464-g_Romboutsia
ASV00378-s_ilealis
ASV00423-g_Romboutsia
ASV00010-s_jejuni
ASV00358-o_Saccharimonadales
ASV00355-g_Campylobacter
ASV00343-s_ilealis
ASV00268-g_Romboutsia
ASV00265-s_ilealis
ASV00263-g_Romboutsia
ASV00224-s_ilealis
ASV00223-g_Romboutsia
ASV00195-g_Romboutsia
ASV00217-s_ilealis
ASV00205-g_Romboutsia
ASV00048-s_magna
ASV00004-s_johnsonii
ASV00043-s_jejuni
-2.5
0.0
2.5
LDA SCORE (log 10)

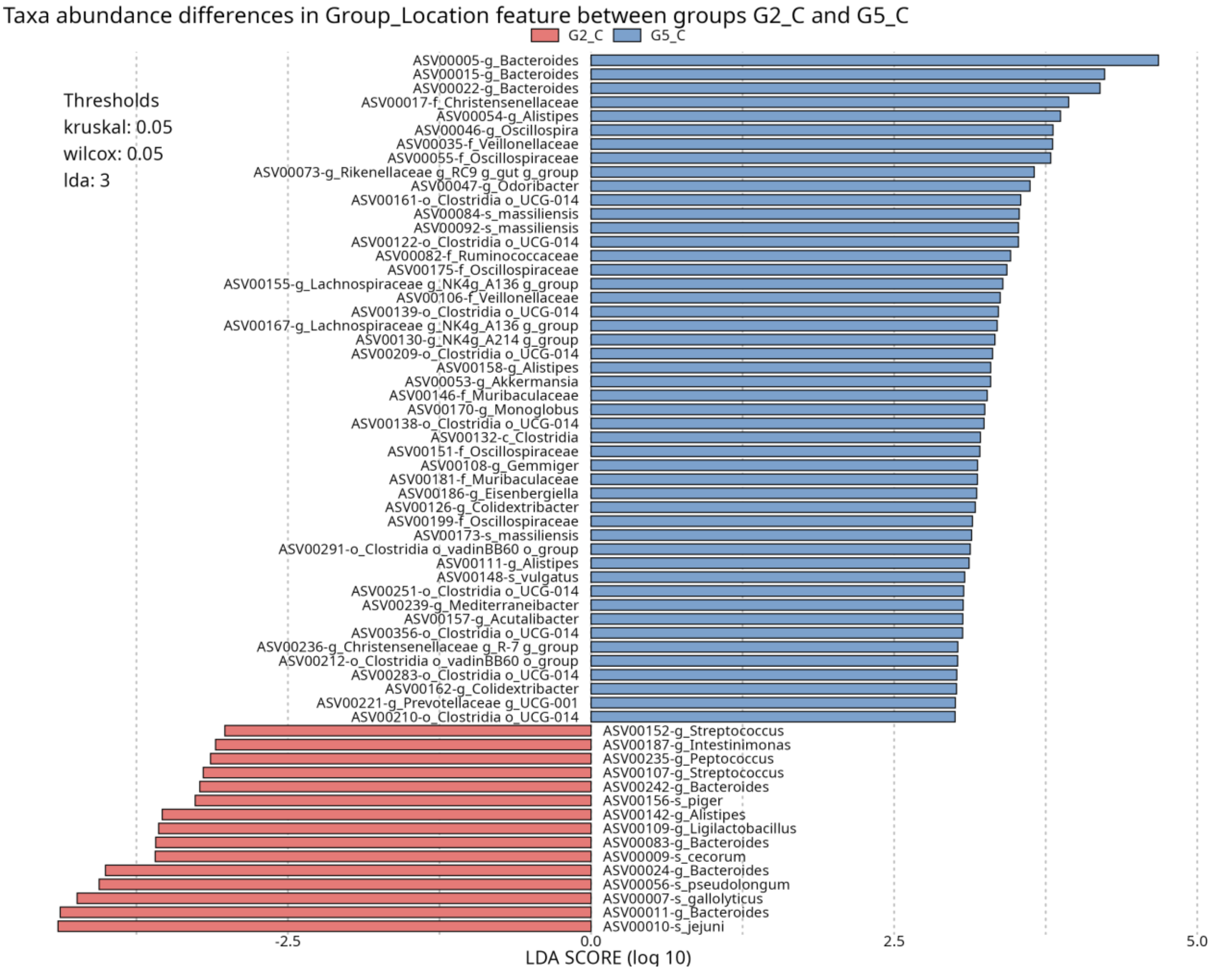
Taxa abundance differences in Group_Location feature between groups G2_C and G5_C
G2_C
G5_C
Thresholds
kruskal: 0.05
wilcox: 0.05
lda: 3
ASV00005-g_Bacteroides
ASV00015-g_Bacteroides
ASV00022-g_Bacteroides
ASV00017-f_Christensenellaceae
ASV00054-g_Alistipes
ASV00046-g_Oscillospira
ASV00035-f_Veillonellaceae
ASV00055-f_Oscillospiraceae
ASV00073-g_Rikenellaceae g_RC9 g_gut g_group
ASV00047-g_Odoribacter
ASV00161-o_Clostridia o_UCG-014
ASV00084-s_massiliensis
ASV00092-s_massiliensis
ASV00122-o_Clostridia o_UCG-014
ASV00082-f_Ruminococcaceae
ASV00175-f_Oscillospiraceae
ASV00155-g_Lachnospiraceae g_NK4g_A136 g_group
ASV00106-f_Veillonellaceae
ASV00139-o_Clostridia o_UCG-014
ASV00167-g_Lachnospiraceae g_NK4g_A136 g_group
ASV00130-g_NK4g_A214 g_group
ASV00209-o_Clostridia o_UCG-014
ASV00158-g_Alistipes
ASV00053-g_Akkermansia
ASV00146-f_Muribaculaceae
ASV00170-g_Monoglobus
ASV00138-o_Clostridia o_UCG-014
ASV00132-c_Clostridia
ASV00151-f_Oscillospiraceae
ASV00108-g_Gemmiger
ASV00181-f_Muribaculaceae
ASV00186-g_Eisenbergiella
ASV00126-g_Colidextribacter
ASV00199-f_Oscillospiraceae
ASV00173-s_massiliensis
ASV00291-o_Clostridia o_vadinBB60 o_group
ASV00111-g_Alistipes
ASV00148-s_vulgatus
ASV00251-o_Clostridia o_UCG-014
ASV00239-g_Mediterraneibacter
ASV00157-g_Acutalibacter
ASV00356-o_Clostridia o_UCG-014
ASV00236-g_Christensenellaceae g_R-7 g_group
ASV00212-o_Clostridia o_vadinBB60 o_group
ASV00283-o_Clostridia o_UCG-014
ASV00162-g_Colidextribacter
ASV00221-g_Prevotellaceae g_UCG-001
ASV00210-o_Clostridia o_UCG-014
ASV00152-g_Streptococcus
ASV00187-g_Intestinimonas
ASV00235-g_Peptococcus
ASV00107-g_Streptococcus
ASV00242-g_Bacteroides
ASV00156-s_piger
ASV00142-g_Alistipes
ASV00109-g_Ligilactobacillus
ASV00083-g_Bacteroides
ASV00009-s_cecorum
ASV00024-g_Bacteroides
ASV00056-s_pseudolongum
ASV00007-s_gallolyticus
ASV00011-g_Bacteroides
ASV00010-s_jejuni
-2.5
0.0
2.5
5.0
LDA SCORE (log 10)

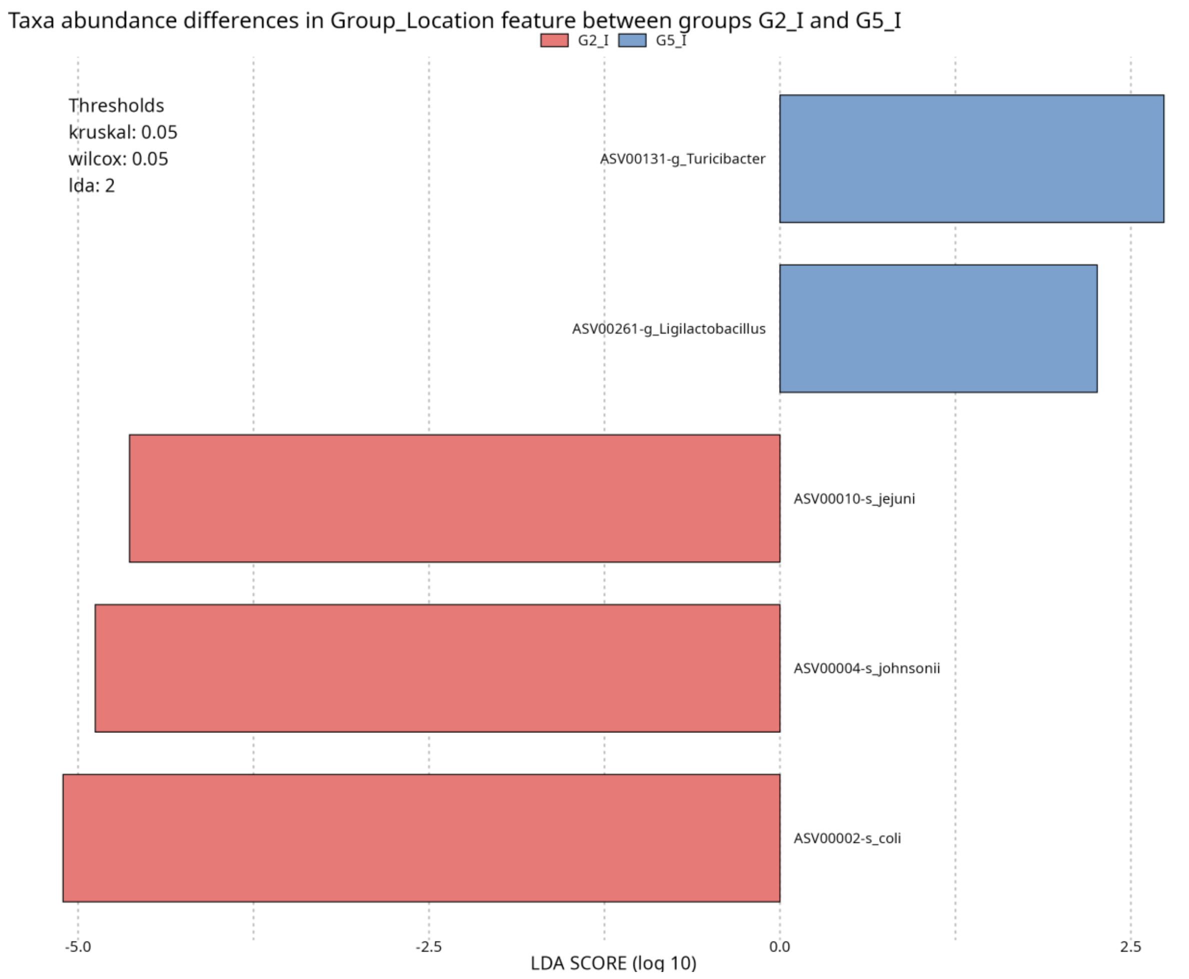
Taxa abundance differences in Group_Location feature between groups G2_I and G5_I
G2_I
G5_I
Thresholds
kruskal: 0.05
wilcox: 0.05
lda: 2
ASV00131-g_Turicibacter
ASV00261-g_Ligilactobacillus
ASV00010-s_jejuni
ASV00004-s_johnsonii
ASV00002-s_coli
-5.0
-2.5
0.0
2.5
LDA SCORE (log 10)

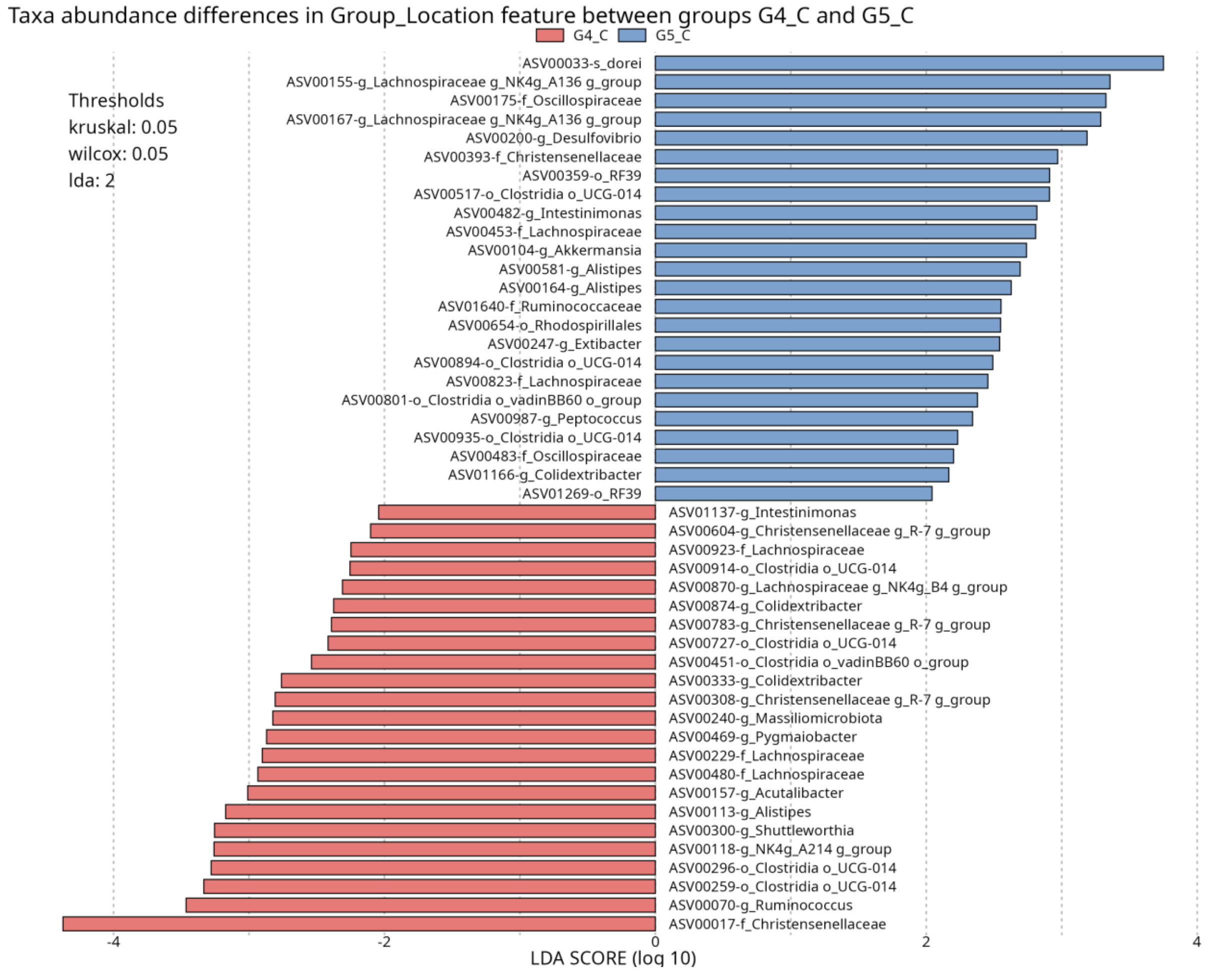
Taxa abundance differences in Group_Location feature between groups G4_C and G5_C
G4_C
G5_C
Thresholds
kruskal: 0.05
wilcox: 0.05
lda: 2
ASV00033-s_dorei
ASV00155-g_Lachnospiraceae g_NK4g_A136 g_group
ASV00175-f_Oscillospiraceae
ASV00167-g_Lachnospiraceae g_NK4g_A136 g_group
ASV00200-g_Desulfovibrio
ASV00393-f_Christensenellaceae
ASV00359-o_RF39
ASV00517-o_Clostridia o_UCG-014
ASV00482-g_Intestinimonas
ASV00453-f_Lachnospiraceae
ASV00104-g_Akkermansia
ASV00581-g_Alistipes
ASV00164-g_Alistipes
ASV01640-f_Ruminococcaceae
ASV00654-o_Rhodospirillales
ASV00247-g_Extibacter
ASV00894-o_Clostridia o_UCG-014
ASV00823-f_Lachnospiraceae
ASV00801-o_Clostridia o_vadinBB60 o_group
ASV00987-g_Peptococcus
ASV00935-o_Clostridia o_UCG-014
ASV00483-f_Oscillospiraceae
ASV01166-g_Colidextribacter
ASV01269-o_RF39
ASV01137-g_Intestinimonas
ASV00604-g_Christensenellaceae g_R-7 g_group
ASV00923-f_Lachnospiraceae
ASV00914-o_Clostridia o_UCG-014
ASV00870-g_Lachnospiraceae g_NK4g_B4 g_group
ASV00874-g_Colidextribacter
ASV00783-g_Christensenellaceae g_R-7 g_group
ASV00727-o_Clostridia o_UCG-014
ASV00451-o_Clostridia o_vadinBB60 o_group
ASV00333-g_Colidextribacter
ASV00308-g_Christensenellaceae g_R-7 g_group
ASV00240-g_Massiliomicrobiota
ASV00469-g_Pygmaiobacter
ASV00229-f_Lachnospiraceae
ASV00480-f_Lachnospiraceae
ASV00157-g_Acutalibacter
ASV00113-g_Alistipes
ASV00300-g_Shuttleworthia
ASV00118-g_NK4g_A214 g_group
ASV00296-o_Clostridia o_UCG-014
ASV00259-o_Clostridia o_UCG-014
ASV00070-g_Ruminococcus
ASV00017-f_Christensenellaceae
-4
-2
0
2
4
LDA SCORE (log 10)

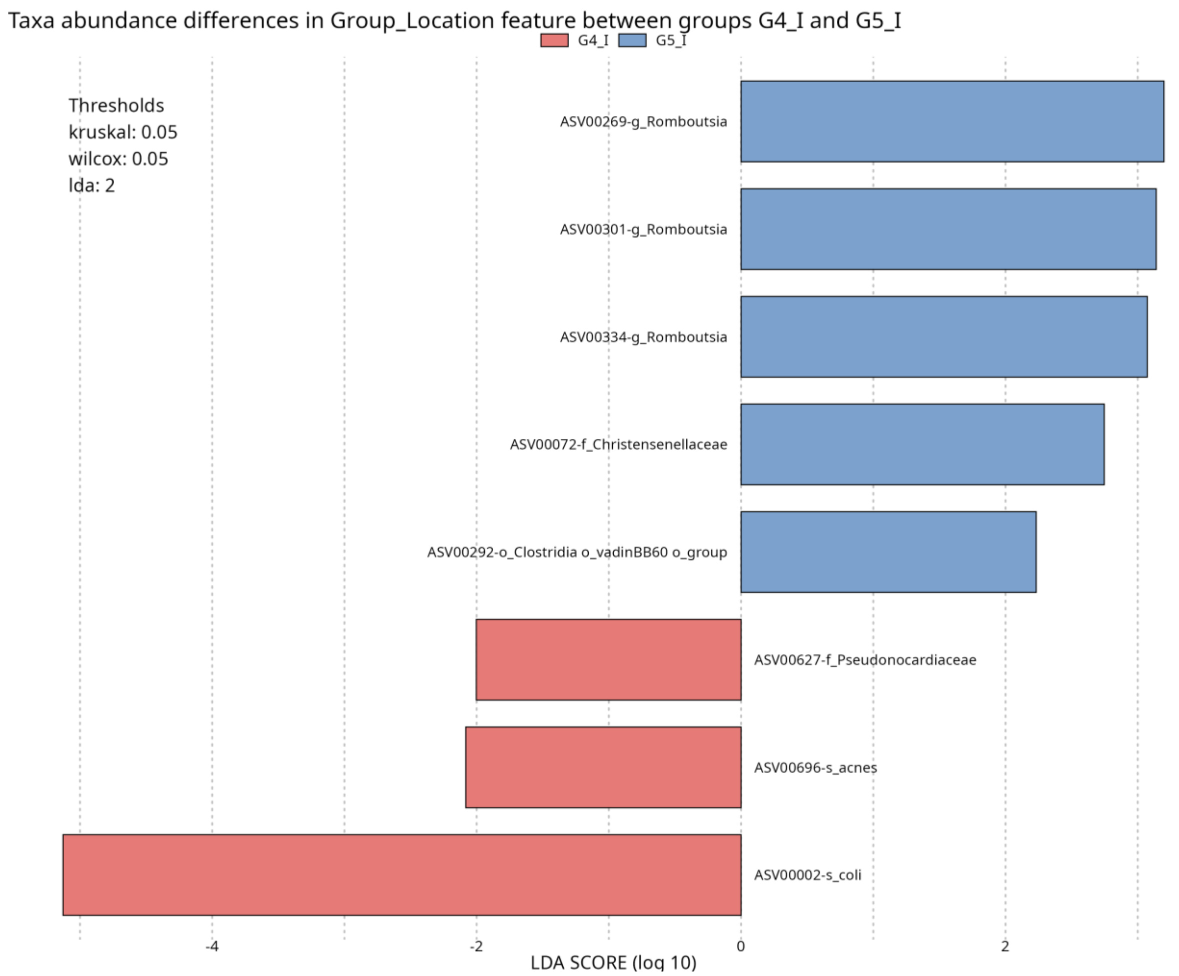
Taxa abundance differences in Group_Location feature between groups G4_I and G5_I
G4_I
G5_I
Thresholds
kruskal: 0.05
wilcox: 0.05
lda: 2
ASV00269-g_Romboutsia
ASV00301-g_Romboutsia
ASV00334-g_Romboutsia
ASV00072-f_Christensenellaceae
ASV00292-o_Clostridia o_vadinBB60 o_group
ASV00627-f_Pseudonocardiaceae
ASV00696-s_acnes
ASV00002-s_coli
-4
-2
0
2
LDA SCORE (log 10)
