# Supplementary material for: A Paired Ileum–Caecum Study of the Goose Gut Microbiome in Response to Fattening Protocol and Postbiotic Supplementation
Source: Animals (Basel). 2026 Oct 8;16(19):3153. doi: 10.3390/ani16193153 (PMC13663337; doi:10.3390/ani16193153)
Supplement: Supplementary file 1 [file animals-16-03153-s001.zip › Supplementary Figure S2 and S3_pcoa_ileum_caecum_Animals.pdf]

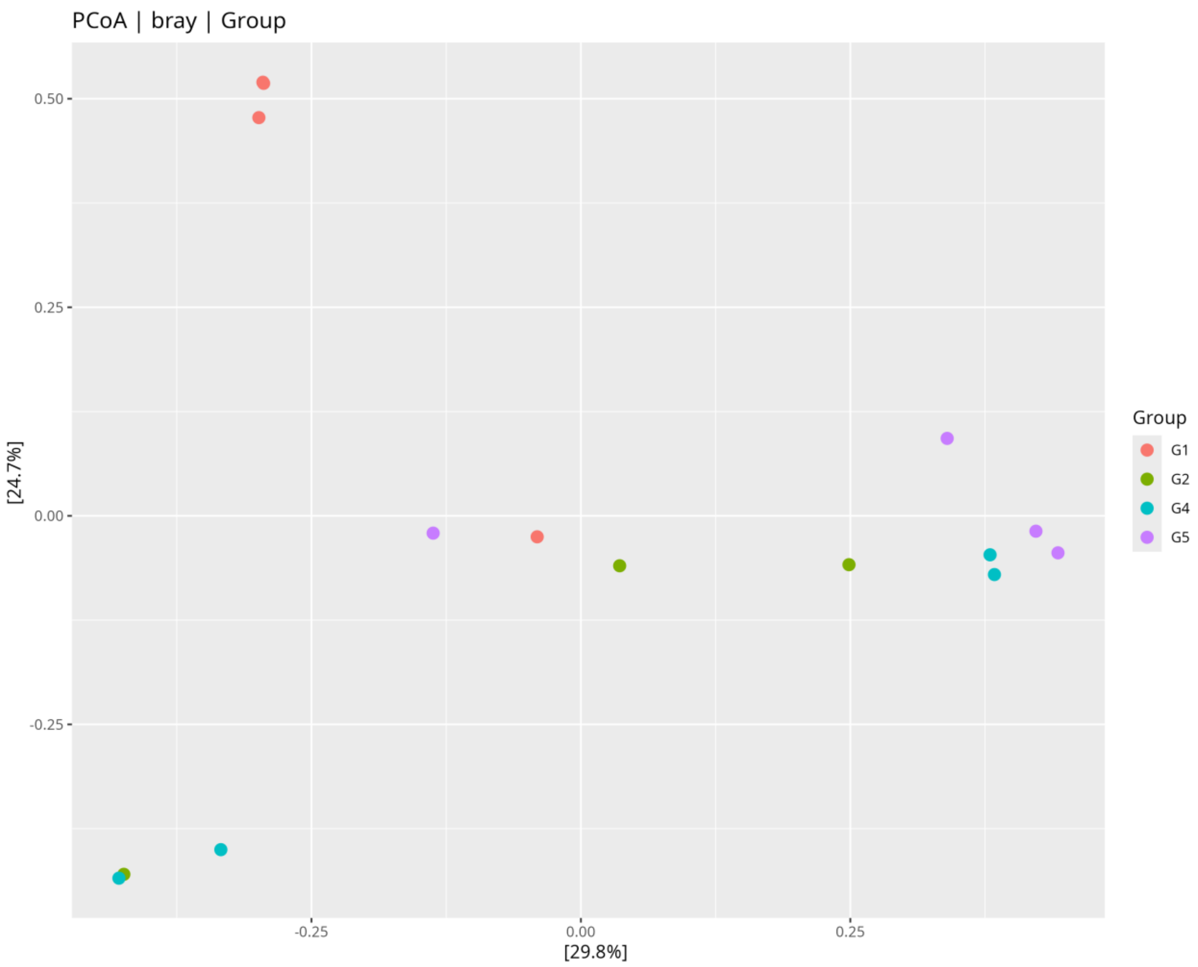

Supplementary Figure S2: Beta diversity plot of Ileum samples (method: PCoA distance calc: bray)

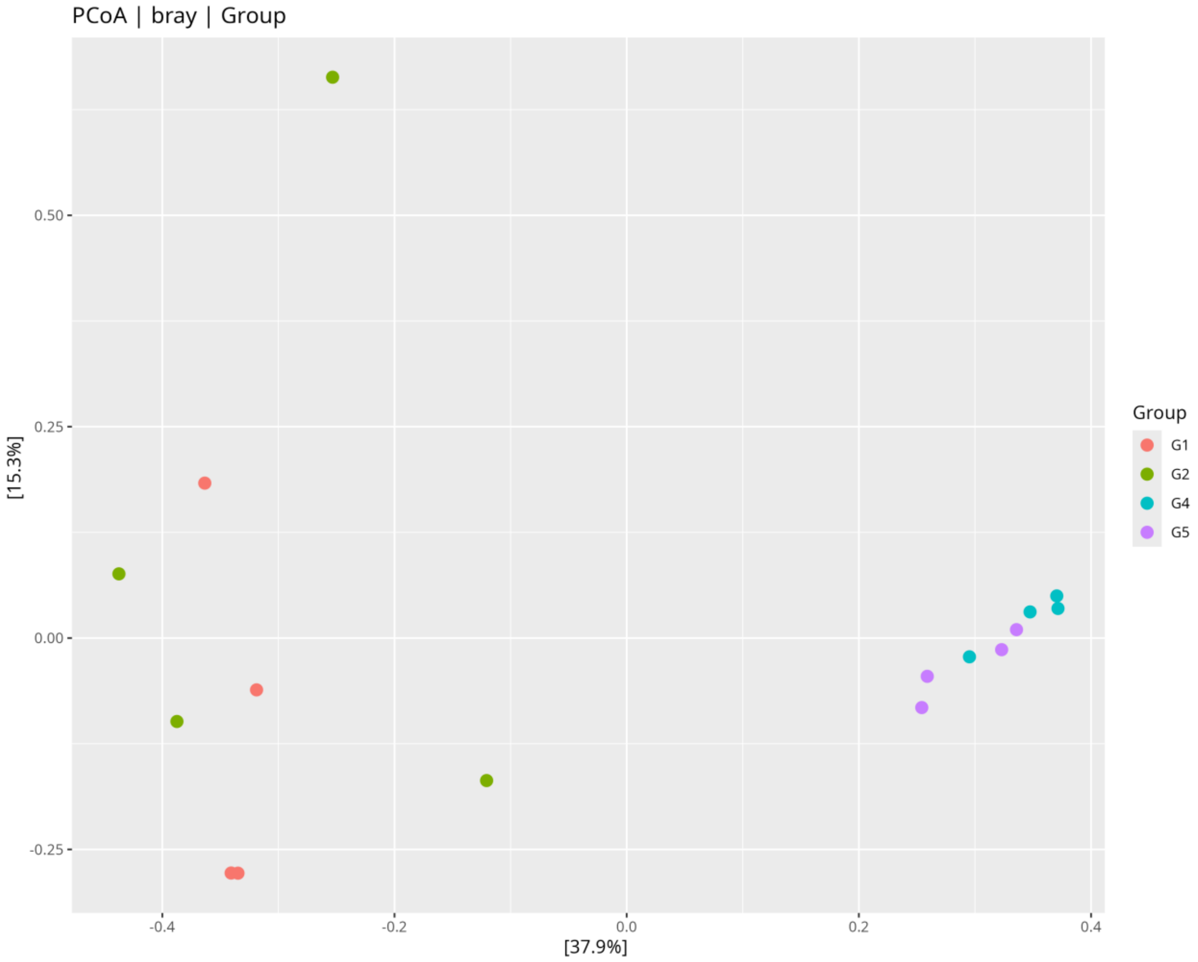

Supplementary Figure S3: Beta diversity plot of Caecum samples (method: PCoA distance calc: bray)
